# Protecting and Diversifying the Germline

**DOI:** 10.1534/genetics.117.300208

**Published:** 2018-02-25

**Authors:** Ryan J. Gleason, Amit Anand, Toshie Kai, Xin Chen

**Affiliations:** * Department of Biology, Johns Hopkins University, Baltimore, Maryland 21218; † Temasek Life Sciences Laboratory, National University of Singapore, Singapore 11760; ‡ Graduate School of Frontier Biosciences, Osaka University, Suita 565-0871, Japan

**Keywords:** oogenesis, spermatogenesis, germline stem cells, somatic gonadal cells, mitosis, meiosis, signaling pathways, piRNA, transcription, chromatin regulator, epigenetics, FlyBook

## Abstract

Gametogenesis represents the most dramatic cellular differentiation pathways in both female and male flies. At the genome level, meiosis ensures that diploid germ cells become haploid gametes. At the epigenome level, extensive changes are required to turn on and shut off gene expression in a precise spatiotemporally controlled manner. Research applying conventional molecular genetics and cell biology, in combination with rapidly advancing genomic tools have helped us to investigate (1) how germ cells maintain lineage specificity throughout their adult reproductive lifetime; (2) what molecular mechanisms ensure proper oogenesis and spermatogenesis, as well as protect genome integrity of the germline; (3) how signaling pathways contribute to germline-soma communication; and (4) if such communication is important. In this chapter, we highlight recent discoveries that have improved our understanding of these questions. On the other hand, restarting a new life cycle upon fertilization is a unique challenge faced by gametes, raising questions that involve intergenerational and transgenerational epigenetic inheritance. Therefore, we also discuss new developments that link changes during gametogenesis to early embryonic development—a rapidly growing field that promises to bring more understanding to some fundamental questions regarding metazoan development.

GAMETOGENESIS produces the only cell types within an organism that contribute genetic, as well as epigenetic, material to the offspring. Germ cells are distinct from the mortal somatic cells in their ability to differentiate into gametes that regain totipotency to produce an entire organism upon fertilization (Cinalli *et al.* 2008). Gametes undergo an extraordinary cellular differentiation process to produce morphologically and functionally distinct gametes, *i.e.*, oocytes and sperm. Studying gametogenesis in *Drosophila* allows us to follow the linear organization of germ cells in adult ovaries and testes. Such organization ensures that all stages of oogenesis and spermatogenesis can be recognized in a highly orchestrated manner (Figure 1) (Fuller 1993; Spradling 1993).

**Figure 1:**
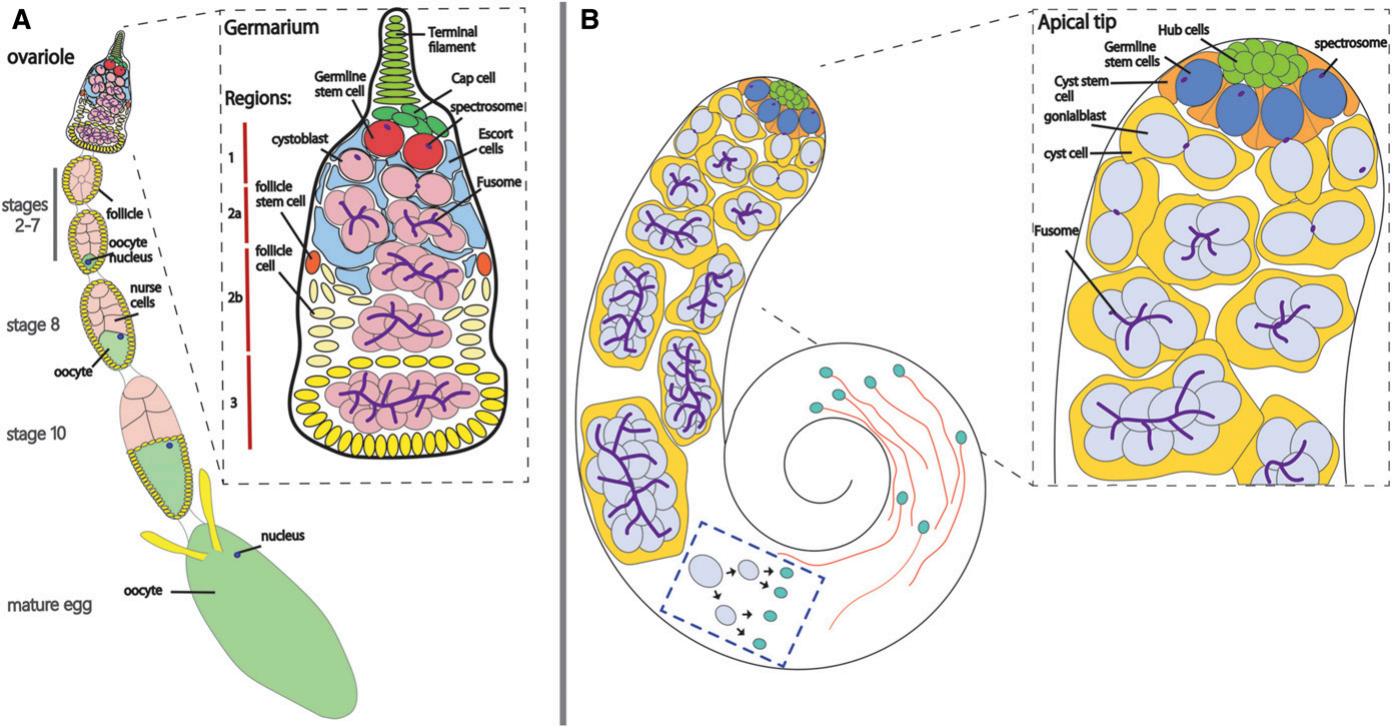
Anatomy of *Drosophila* female and male gonads. (A) Anatomy of germarium and oogenesis. The *Drosophila* ovaries are made up of 16–20 tubule structures, called ovarioles, that resemble linear assembly lines of progressively differentiating egg chambers to produce eggs. The germanium, designated by the dashed rectangular outline, where the egg chamber originates, is located at the anterior tip of each ovariole. The germarium consists of the GSC niche and the proliferative germ cells that remain active, producing eggs throughout adulthood. At the anterior tip of each germanium resides the niche, which consists of a stack of 8–10 postmitotic somatic cells, called the terminal filament (light green), five to seven squamous epithelial cells, and cap cells (dark green) that literally cap the underlying two to three GSCs (red). Female GSCs divide asymmetrically such that the anteriorly positioned daughter cell remains in contact with the cap cells and maintains GSC identity, while the posteriorly displaced daughter cell leaves the niche and differentiates into cystoblasts (CBs) (pink). Following the asymmetric cell division, the daughter CB undergoes four rounds of synchronous mitotic cell divisions with incomplete cytokinesis to give rise to 16 interconnected cystocytes (pink). During early germ cell development, early germ cells associate intimately with neighboring somatic cells, including escort cells and follicle cells (yellow). Interspersed between the GSCs are four to six escort cells (blue cells), which cover most of the GSC and dividing CBs, isolating early germ cells from each other, but not from the cap cells. Next, the interconnected germ cell cyst associates with another somatic cell type, the somatic follicle cells. These somatic follicle cells are derived from two somatic follicle stem cells (FSCs) (orange), which are maintained at the boundary between escort cells and the follicle cells. When the 16-cell cyst is surrounded by follicle cells, it becomes an egg chamber, buds from the germanium, and continues to mature (Davring and Sunner 1973). One of the 16 cells will progress through meiosis and develop into the oocyte, while the other cells will develop into polyploid nurse cells that will support oocyte growth. A single egg chamber consists of the single oocyte connected to 15 nurse cells via a system of intercellular bridges and a surrounding monolayer of up to 650 somatic follicle cells (King 1970; Spradling 1993). The nurse cells deliver their cytoplasm into the oocytes and undergo apoptosis during the latest stage of oogenesis to produce a mature egg (Foley and Cooley 1998). Meiotic divisions in the egg are only accomplished after sperm entry, leading to one female pronucleus and three polar bodies, which subsequently undergo degeneration. The female and male pronuclei appose each other, followed by fusion, which labels the formation of a zygote and the onset of a new life cycle. (B) Anatomy of testis and spermatogenesis. The adult testis of *Drosophila melanogaster* is a pair of coiled tubes ∼2 mm in length, each composed of a single stem cell niche at the apical end designated by the dashed gray outline (Hardy *et al.* 1979). The apical cells are assembled into a centrally located structure having GSCs (dark blue), and CySCs (orange) are radially positioned around a cluster of 10–12 small densely packed somatic cells called the hub (Green). Six to nine GSCs are arranged around the hub, while two CySCs fully envelope one GSC such that GSC-to-GSC contact never occurs. Spermatogenesis initiates with the asymmetric division of the GSC to produce one self-renewed daughter cell and a gonialblast (GB) cell (light blue). Upon division, the GB is displaced from the hub and undergoes four transit-amplifying divisions with incomplete cytokinesis, generating a cyst of interconnected germ cells joined by cytoplasmic bridges (light blue). After transit-amplification, the cyst of 16 interconnected spermatogonia synchronously undergoes meiotic DNA synthesis. During meiotic prophase I as spermatocytes, each cell grows ∼25-fold and initiates a robust gene expression program that enables meiotic division and spermatid differentiation. After two meiotic divisions, 64 haploid spermatids are produced, as designated by the dashed blue outline.

Throughout oogenesis and spermatogenesis, germ cells are closely associated with somatic gonadal cells. In females, germline stem cells (GSCs) first interact with escort cells, followed by the follicle stem cell (FSC) lineage. FSC homeostasis depends on an epithelial niche structure that involves migration of FSCs across the ovariole (Nystul and Spradling 2007, 2010) (Figure 1A). In males, each GSC is enclosed by two cyst stem cells (CySCs). Through asymmetric cell division (Cheng *et al.* 2011), CySCs self-renew and give rise to differentiated cyst cells, which never divide again. Two cyst cells encapsulate synchronously dividing and differentiating germ cells, and form a distinct germ cell cyst (Figure 1B). Increasing evidence demonstrates that somatic gonadal cells are not only support cells, but also play important roles in instructing germ cell differentiation and maintaining germline identity. *Drosophila* gametogenesis studies have greatly benefited from enriched genetics tools, including many cell type- and stage-specific Gal4 drivers (Table 1) for performing targeted knockdown, rescue, or overexpression experiments.

**Table 1:** Cell-type-specific Gal4 drivers in *Drosophila* gonads

Cell-Type Expression	Name	Description	References
Male germline			
Germline expression	nos-Gal4-VP16	GSCs and early germline cysts	Van Doren *et al.* (1998)
	bam-Gal4-VP16	Initiates expression during transit-amplification divisions and expressed in early spermatocytes	Chen and McKearin (2003b)
	vas-Gal4	Most germline cells	Zhao *et al.* (2013)
Hub cells	upd-Gal4	Hub cells	Zeidler *et al.* (1999)
	hh-Gal4	Hub cells	Tanimoto *et al.* (2000)
	fasIII-Gal4	Hub cells	Wolfstetter and Holz (2012)
CySCs and somatic cells	tj-Gal4	CySCs, early cyst cells, and hub cells	Hayashi *et al.* (2002)
	C587-Gal4	CySCs, early cyst cells, and hub cells	Kai and Spradling (2003), Zhu and Xie (2003)
	ptc-Gal4	CySCs and cyst cells	Tazuke *et al.* (2002)
	eya-gal4	CySCs and cyst cells, weakly expressed in hub cells	Leatherman and Dinardo (2008)
Somatic cells	arm-Gal4	Most somatic cells including Hub cells, CySCs, and cyst cells	Sanson *et al.* (1996)
Germline and somatic cells	Hsp83-Gal4	Ubiquitously expressed	Arama *et al.* (2003)
Female germline			
Germline expression	nos-Gal4 [pBac(GreenEye.nosGal4-VP16)]	Stem cells, young egg chambers, and increased expression at stage 5	Holtzman *et al.* (2010)
	nos-Gal4-VP16	Stem cells, young egg chambers, and increased expression at stage 5	Van Doren *et al.* (1998)
	Maternal Triple Driver (MTD)-Gal4	Uniform expression in the germarium and throughout oogenesis, including GSCs	Petrella *et al.* (2007)
	bam-Gal4-VP16	Germ cell expression starting at the two-cell cyst stage or CB cells	Chen and McKearin (2003b)
	pCOG-Gal4-VP16	Moderate levels throughout oogenesis	Rorth (1998)
Escort cells	C587-Gal4	Escort cells	X. Song *et al.* (2004)
Terminal filament and Cap cells	bab1-Gal4	Terminal filament and cap cells	Cabrera *et al.* (2002)
	hh-Gal4	Terminal filament and cap cells	Tanimoto *et al.* (2000)
Follicle stem cells	109-30-Gal4	Follicle stem cells and early follicle lineage	Hartman *et al.* (2010)
Follicle cells	Cb16-Gal4	All follicle cells starting in germarium	Ward *et al.* (2002)
Somatic cells	tj-Gal4	All follicle, follicle stem cells, escort cells, and cap cells	Hayashi *et al.* (2002)
Ubiquitous expression	tub-Gal4	Ubiquitous expression in all cells	Lee and Luo (1999)

It has been demonstrated that transcriptional changes at both local and global levels are robust throughout gametogenesis. Epigenetic mechanisms that modify chromatin state without altering primary DNA sequences have profound influence on regulating dynamic transcriptome changes in germ cells. Epigenetic regulation could act through modifications of DNA-associated proteins and/or RNAs, resulting in structural changes of chromatin or recruitment of effector proteins or RNAs, and leading, in turn, to activation or repression of target gene(s). The basic unit of chromatin is called a nucleosome, which contains 147 bp of DNA wrapped around a histone octamer composed of two copies each of H3, H4, H2A, and H2B—the core histones. The major epigenetic mechanisms known to orchestrate cell fate and function include (1) DNA methylation; (2) nucleosome repositioning driven by chromatin remodeling factors; (3) post-translational modifications (PTMs) of histones (*e.g.*, methylation, acetylation, phosphorylation, and ubiquitination, etc.); (4) incorporation of histone variants; and (5) noncoding RNA-mediated chromatin regulation, including piRNA- and microRNA-mediated mechanisms.

As the germline genome is inherited across generations, it is threatened by transposons—genetic mobile elements parasitizing the genome. Transposons are discrete, autonomous, DNA sequences capable of moving from one place to another throughout the genome, or simply increasing their copies in the genome. Transposons constitute almost 23% of the *Drosophila* genome (Lander *et al.* 2001; Huang *et al.* 2012). In order to spread throughout the population, the transposon targets the germline genome, which carries the genetic information from one generation to another for species continuity. Active mobilization of transposons results in insertional mutations, leading to massive destruction of the genome, as well as sterility. Metazoans have evolved a small RNA-based repression system to combat a wide variety of transposons in gonads, called the PIWI-interacting RNA (piRNA) pathway, whose basic function and genes thereof are conserved from the lower invertebrates to mammals (Lim and Kai 2015).

Studies in recent years have shed light on how different mechanisms regulate extensive cellular differentiation during gametogenesis and protect germline identity. In this chapter, we focus on the most recent discoveries of epigenetic regulation and protection of the genome during *Drosophila* oogenesis and spermatogenesis. We start by discussing how known epigenetic mechanisms maintain GSC identity and activity. We next proceed to an examination of their roles in controlling mitotic germ cell proliferation, proper mitosis-to-meiosis transition, and meiotic maturation. Finally, the developing field of intergenerational and transgenerational epigenetic inheritance is explored.

## Mechanisms Regulating GSC Self-Renewal *vs.* Differentiation

### DNA methylation and demethylation

DNA methylation is a widely conserved epigenetic mechanism that functions through the covalent and heritable modification of genomic DNA at both cytosine and adenine residues (Suzuki and Bird 2008; Luo *et al.* 2015). DNA methylation of the fifth position of cytosine (5-methylcyosine, 5mC) is established and maintained by a conserved family of enzymes called DNA methyltransferases (DNMTs), which have been found to function in transcriptional silencing of promoters, transposable elements, and other repetitive sequences in most plant, animal, and fungal species (Wu and Zhang 2014). DNMTs are divided into three subfamilies based on sequence conservation and function. DNMT3 functions in the *de novo* methylation of cytosine. DNMT1 maintains DNA methylation postreplication on the newly synthesized DNA strands, and DNMT2 exhibits a weak catalytic activity on DNA compared to DNMT3 (Hermann *et al.* 2003). DNMT2-mediated methylation of multiple transfer ribonucleic acids (tRNAs) has also been documented (Goll *et al.* 2006; Schaefer *et al.* 2010). *Drosophila* belongs to the “DNMT2 only” category of organisms based on loss of the canonical DNA methyltransferases (DNMT1 and DNMT3), and retention of the DNMT2 homolog *DNMT2/Mt2*. In addition, 5mC levels in *Drosophila* have been found to be very low compared to other organisms (Lyko *et al.* 2000a; Phalke *et al.* 2009; Krauss and Reuter 2011; Raddatz *et al.* 2013; Capuano *et al.* 2014; Takayama *et al.* 2014; Zhang *et al.* 2015). For example, in mammalian DNA, between 2 and 10% of all cytosine residues are modified to 5mC, whereas *Drosophila* DNA contains only 0.1–0.6% of modified 5mC of all cytosine residues (Gowher *et al.* 2000; Zemach *et al.* 2010). While the mechanism by which *DNMT2* functions in the germline remains obscure, recent studies have revealed that *DNMT2* is involved in multiple processes, including sister chromatids' segregation in the male germline, retrotransposon silencing in the early embryo, and gene silencing (Phalke *et al.* 2009; Yadlapalli and Yamashita 2013). Expression of *DNMT2* was first observed in the ovaries, as well as during early embryogenesis through RNA *in situ* hybridization, and more recently in the male germline (Lyko *et al.* 2000b; Gan *et al.* 2010a). *DNMT2* function was found to be necessary for proper segregation of X and Y sister chromatids during asymmetric male GSC divisions (Yadlapalli and Yamashita 2013).

Until recently, DNA methylation of the sixth position of Adenine (6mA) was thought to be restricted to bacteria, archaea, protists, and fungi (Wion and Casadesus 2006). However, recent studies have identified 6mA to be present in 0.07–0.001% of all adenine residues in the *Drosophila* genome during early- and late-stage embryogenesis, respectively (Zhang *et al.* 2015). Demethylation of 6mA is regulated by the *Drosophila* Tet homolog, DNA 6mA demethylase (*Dmad*), during embryogenesis and oogenesis. During oogenesis, loss of *Dmad* results in an increase of 6mA in the ovaries and accumulation of GSC-like cells. On the other hand, overexpression of *Dmad* leads to a significant loss of germ cells, including GSCs. Furthermore, *Dmad*-mediated 6mA demethylation correlates with transposon suppression, indicating that *Dmad* actively removes 6mA to suppress transposon expression (Zhang *et al.* 2015). These results are consistent with the role *Dmad* plays in the demethylation of 6mA to promote GSC differentiation during oogenesis. Together, these results indicate that 5mC has a limited or spatiotemporally specific role in *Drosophila*, likely independent of *DNMT2*/*Mt2* whose enzymatic role is yet to be defined. On the other hand, recent data demonstrate important roles of 6mA and its demethylase *Dmad* in *Drosophila* oogenesis and embryogenesis, even though the corresponding methyltransferase has not been characterized. Given the clear biological functions of 5mC in mammals, it is possible that fly and mammals use distinct DNA methylation mechanisms for their epigenomes.

### Chromatin remodeling factors

The chromatin structure of GSCs and somatic stem cells (SSCs) is regulated by ATP-dependent chromatin remodeling enzymes in both males and females in order to maintain self-renewal and prevent differentiation. These enzymes utilize the energy of ATP hydrolysis to establish and maintain a particular chromatin state during development. The different subfamilies of chromatin-remodeling enzymes catalyze a remarkable range of chromatin modifications that include histone exchange, translocating the histone octamer and changing the conformation of nucleosomal DNA (Narlikar *et al.* 2013). Common across all ATP-dependent chromatin remodeling enzymes is the ATPase subunit belonging to the helicase superfamily 2 (SNF2) (Eisen *et al.* 1995). This SNF2 family of proteins can be further classified on the basis of distinct domains conserved among the subfamilies, such as the bromodomain shared by the SWI2/SNF2 (SWItch/Sucrose NonFermentable) family, the chromodomain shared by the CHD (Chromodomain-Helicase-DNA-binding protein) family, and the SANT domain shared by the ISWI (Imitation SWI) family (Hota and Bruneau 2016).

#### Imitation switch (ISWI):

In *Drosophila*, ISWI serves as an ATP-dependent motor that governs transcriptional regulation through catalyzing changes in nucleosomal assembly and composition (Deuring *et al.* 2000; Badenhorst *et al.* 2002; Corona *et al.* 2002). In both males and females, ISWI is essential for GSC maintenance, suggesting a common epigenetic mechanism employed by both sexes to maintain a chromatin configuration for stem cell maintenance. In females, ISWI is present at high levels in all cell types, including GSCs and FSCs (Xi and Xie 2005). Mitotic recombination techniques (Xu and Rubin 1993) were used to generate marked *iswi* mutant GSC clones, most of which were lost from the niche owing to premature differentiation. Similar to ISWI, bone morphogenetic protein (BMP) signal transduction is essential for GSC maintenance. Upon signal transduction, the BMP signaling cascade is mediated by phosphorylated MAD (pMAD), which activates the target *Daughters against dpp* (*Dad*) transcription, and results in transcriptional repression of the differentiation marker *bag of marbles* (*bam*) (Chen and McKearin 2003a; X. Song *et al.* 2004). Since cystoblasts (CBs) do not receive enough BMP ligand, they begin the differentiation process by the increased expression of *bam* (Y. Li *et al.* 2009). Significant premature upregulation of *bam* was found in *iswi* mutant GSCs when compared to wild-type GSC clones. *Dad* transcription was also aberrantly regulated in the absence of *iswi*. These results demonstrate that ISWI maintains GSC self-renewal through BMP signaling-mediated gene expression.

#### Nucleosome remodeling factor (NURF) complex:

In *Drosophila*, ISWI is a component of three chromatin remodeling complexes, including NURF (NUcleosome Remodeling Factor), ACF (ATP-utilizing Chromatin assembly and remodeling Factor), and CHRAC (CHRomatin Accessibility Complex). In males, GSC self-renewal is specifically regulated by the NURF complex (Cherry and Matunis 2010), which is composed of ISWI, NURF301, NURF55, and NURF38. Similar to the *iswi* mutant female GSC phenotype, inactivation of *iswi* and *Nurf301* leads to loss of male GSCs from premature differentiation by precocious expression of Bam. Clonal analysis revealed that *Nurf301* mutant CySCs, similar to GSCs, are lost rapidly as a result of premature differentiation.

Similar to the NURF complex, JAK/STAT signaling is also required for the maintenance of both GSCs and CySCs. In the male, the hub cells secrete the signaling ligand Unpaired (Upd) to support stem cell self-renewal of both GSCs and CySCs, as well as adhesion of GSCs to the hub cells (Kiger *et al.* 2001; Tulina and Matunis 2001; Leatherman and Dinardo 2008, 2010). Loss-of-function of either the Janus kinase (JAK), encoded by *hopscotch* (*hop*), or the signal transducer and activator of transcription (STAT), encoded by *Stat92E*, in the germline leads to rapid loss of GSCs and early germ cells (Kiger *et al.* 2001; Tulina and Matunis 2001). Consistent with the role of the JAK/STAT pathway in GSC self-renewal, ectopic expression of Upd in early germ cells leads to a dramatic increase in the number of GSC-like cells with a concomitant decrease in the number of cells undergoing differentiation. To test whether the NURF complex regulates the maintenance of GSCs and CySCs through mediating JAK-STAT signaling from the niche, JAK-STAT activity was monitored in *Nurf301* null clones by measuring STAT92E expression levels. Loss of *Nurf301* resulted in decreased STAT92E, suggesting that *Nurf301* promotes the maintenance of GSCs, at least in part, through positively regulating the JAK-STAT pathway. Furthermore, *suppressor of cytokine signaling 36E* (*Socs36E*) is a conserved target of the JAK/STAT pathway in CySCs that functions in a negative feedback loop by downregulating JAK/STAT activity (Issigonis *et al.* 2009). Similar to loss of JAK/STAT signaling, downregulation of NURF301 partially rescued the *Socs36E* phenotype. These studies highlight that the chromatin remodeling complex, the NURF complex, functions as a positive regulator of JAK/STAT signaling in both GSCs and CySCs in the testis.

Recent studies have also revealed that the ecdysone steroid hormone pathway acts through the NURF complex in female GSCs and in male CySCs (Ables and Drummond-Barbosa 2010; Li *et al.* 2014). The ecdysone receptor (EcR) is expressed throughout the ovary in multiple cell types (Buszczak *et al.* 1999). Upon binding of ecdysone to EcR, EcR dimerization occurs with Ultraspiracle (Usp), initiating a transcriptional cascade that includes *E74*, *E75*, and *broad* (*br*) as targets (Riddiford *et al.* 2000). Analysis of GSC clones homozygous for *usp* and *E74* in female and temperature-sensitive alleles of ecdysone and *EcR* demonstrated that ecdysone signaling promotes GSC maintenance. Interestingly, genetic interactions were discovered between the NURF complex genes *iswi* and *Nurf301* and the Ecdysone pathways genes *usp* and *E74*. Additionally, *loss-of-function* mutations in *usp* and *E74* result in reduced levels of nuclear ISWI. As mentioned earlier, *iswi* mutations result in aberrant BMP signaling and premature *bam* expression. Consistent with the decrease in ISWI, BMP signaling levels are reduced in *usp* and *E74* null clones. The ecdysone signaling pathway acts with the NURF chromatin remodeling complex to promote female GSC maintenance. Intriguingly, purified NURF physically interacts with EcR in an ecydsone-dependent manner, and expression of EcR target genes is significantly reduced in *Nurf* mutants, suggesting that Nurf is a coactivator of EcR (Badenhorst *et al.* 2002).

In the male, ecdysone signaling components are expressed in both hub cells and the CySC lineage, and they are required for CySC maintenance (Li *et al.* 2014). Loss of ecdysone signaling in CySCs results in loss of GSCs, as well as CySCs, suggesting that EcR signaling contributes to both stem cell populations in the testis. It currently remains unknown whether GSC maintenance requires an ecdysone-dependent or -independent signal from CySCs.

#### Domino (dom):

This SWR1-like ATP-dependent chromatin remodeling factor functions in both male and female gonads for stem cell self-renewal (Xi and Xie 2005; Morillo Prado *et al.* 2013). Unlike the chromatin remodeling factor ISWI, which is essential for GSC self-renewal, DOM is only essential for FSC self-renewal in the female. Clonal analysis of *dom* mutant GSCs reported no change in their division rate. In contrast, female FSCs marked for loss of *dom* demonstrated that DOM specifically controls FSC self-renewal, but not survival (Xi and Xie 2005). In the female, these studies revealed that different stem cell types, GSCs and FSCs, depend on distinct chromatin remodeling factors, ISWI and DOM, respectively, to control their self-renewal.

In the male, clonal analysis revealed that DOM is required cell autonomously for both GSC and CySC maintenance, and may regulate the incorporation of the histone variant H2Av (Morillo Prado *et al.* 2013). H2Av is the *Drosophila* sole homolog of mammalian H2A.Z and H2A.X (Talbert and Henikoff 2010; Baldi and Becker 2013). Although the expression of H2Av is ubiquitous, its function is dispensable for germline and cyst cell differentiation, suggesting a specific role for maintaining the stem cell state in these lineages. H2Av, which is incorporated by SWR1-like remodeling complexes, regulates transcriptional control, formation of heterochromatin boundaries, lineage commitment, and DNA repair throughout development (Henikoff *et al.* 2004; Creyghton *et al.* 2008; Venkatesh and Workman 2015). Dom has been purified from S2 cells as part of a 16-subunit assembly, and this complex has been shown to exchange H2Av *in vitro* (Kusch *et al.* 2004). Because Dom is required for H2Av incorporation, loss of *dom* function reduced H2Av levels in male GSCs. Furthermore, a recent study has highlighted specific roles for a distinct *dom* splicing variant required for the incorporation and removal of H2Av during oogenesis (Borner and Becker 2016). Similar to *dom*, H2Av is required for both male GSC and CySC maintenance independent of the JAK/STAT pathway, and it has been implicated in both transcriptional repression and activation (Morillo Prado *et al.* 2013). Lack of H2Av does not result in global changes in H3K4me3 or H3K27me3 immunostaining pattern. However, it is possible that the *H2Av* mutation disrupts H3K9me2/3-enriched heterochromatin structure in GSCs, as previously shown in somatic cells (Swaminathan *et al.* 2005). Therefore, DOM and H2Av may be required to maintain GSC and CySCs by facilitating repression of differentiation genes and/or maintaining activation of genes necessary for GSC self-renewal. Finally, *dom* mutants can be partially rescued by the human ortholog, SRCAP (Eissenberg *et al.* 2005).

#### Brahma:

Brahma (Brm), a bromodomain protein, is the sole member of the *Drosophila* SWI/SNF-type ATPase chromatin remodeler. It has cell-autonomous, as well as non-cell-autonomous, roles in regulating female GSC self-renewal (Brizuela *et al.* 1994; Elfring *et al.* 1998; Zraly *et al.* 2003; He *et al.* 2014). Brahma is a member of two protein complexes, BAP and PBAP, and it is expressed in all cell types in the germarium and follicle cells. Both complexes share seven subunits, including Brm, and differ in three subunits. OSA is a member of the BAP complex, while Polybromo and BAP170 are members of the PBAP complex (Mohrmann *et al.* 2004). Using both clonal analysis of *brm* mutant and tissue-specific RNAi knockdown, a cell-autonomous role of *brm* in sustaining the GSC population has been revealed. Furthermore, knocking down *brm* in the niche cells showed a non-cell-autonomous role for *brm* in regulating GSC self-renewal. To distinguish whether a specific Brm complex, BAP or PBAP, regulates GSC self-renewal, loss of *osa* and *polybromo/bap180* was tested individually. This revealed that mutations in *polybromo*/*bap180*, rather than *osa*, cause similar GSC loss phenotype. These studies indicate that Brm functions in the PBAP complex for GSC maintenance.

#### Nclb:

A novel chromatin factor encoded by *no child left behind* (*nclb*) specifically regulates male, but not female, GSC maintenance (Casper *et al.* 2011). Nclb is enriched at chromatin regions with active transcription. In *nclb* mutant GSCs, *Stat92E* has decreased transcription or protein accumulation (Casper *et al.* 2011), suggesting that Nclb acts via signaling pathways to determine GSC fate.

### Histones

**Figure 2:**
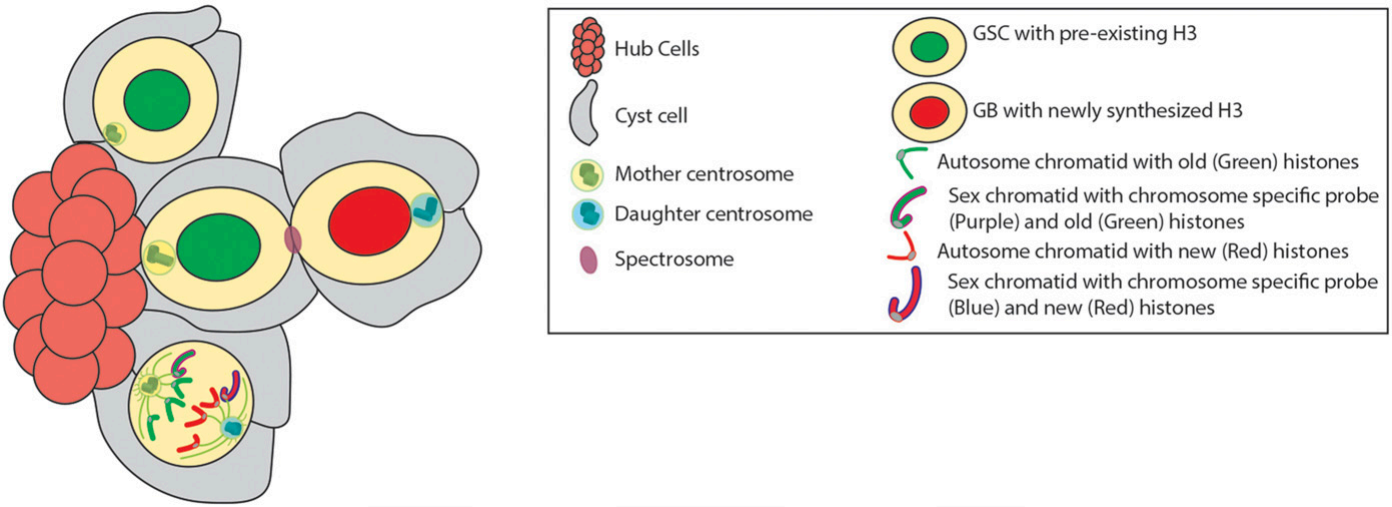
Asymmetric histone inheritance, Nonrandom segregation of sister chromatids, and asymmetric centrosome inheritance during male GSC asymmetric cell division. Investigations into asymmetric cell division using a dual-color labeling strategy to label pre-existing (green) *vs.* newly (red) synthesized canonical histone H3 have revealed that old histone H3 is selectively retained in the self-renewed GSC (green nuclei), whereas newly synthesized H3 is enriched in the differentiating daughter cell (red nuclei). More studies are needed to investigate whether the observed H3 asymmetry occurs at all chromosomes, particular chromosomes, or specific genomic regions. During this division, mother and daughter centrosomes with distinct microtubule nucleating capabilities are also observed to be asymmetric: the mother centrosome (yellow and green) remains in proximity to the stem cell niche, while the daughter centrosome (light-blue and turquoise) migrates to the distal side of the cell leading to a perpendicular spindle orientation relative to the niche and asymmetric centrosome inheritance. Using CO-FISH (Chromosome Orientation Fluorescence *in situ* Hybridization) combined with strand-specific probes to distinguish sister chromatids it has been shown that sex chromosomes (Purple outlined chromatid and Blue outlined chromatid), including both X and Y, exhibit an ∼85:15 biased segregation of sister chromatids during male GSC cell division.

The principal components of epigenetic information, histones, are uniquely distributed with pre-existing (old) histone H3 segregating to the stem cell and newly synthesized (new) H3 localizing to the differentiating daughter cell during *Drosophila* male GSC asymmetric division [Figure 2, (Tran *et al.* 2012)]. The histone variant H3.3, which is incorporated in a replication-independent manner, does not exhibit such an asymmetric pattern. Therefore, it is likely that DNA replication plays an important role in establishing histone asymmetry between sister chromatids. Furthermore, asymmetric H3 inheritance occurs specifically in the asymmetrically dividing GSCs, but not in the symmetrically dividing progenitor germ cells, suggesting that polarized mitotic machinery could contribute to recognizing the sister chromatid asymmetry established by replication. Cellular specificity exhibited by H3 suggests that global asymmetric histone inheritance occurs uniquely in a cell type (GSC) where the mother cell must divide to produce two daughter cells, each with a unique cell fate. However, more research is required to investigate whether the observed H3 asymmetry occurs at all chromosomes, particular chromosomes, or at specific genomic regions. It has also been shown that differential phosphorylation at Threonine 3 of H3 (H3T3P) distinguishes old *vs.* new histones in dividing GSCs. The H3T3P is enriched at the pericentric region and is only detectable from prophase to early anaphase. The tight spatiotemporal regulation of this phosphorylation likely ensures that it acts at the right location and with the precise timing. Misregulation of this phosphorylation, using either a dominant negative mutant or a phosphomimetic form, leads to randomized segregation of old *vs.* new histones, as well as stem cell loss and germline tumors. This finding sheds light on the biological significance of asymmetric histone inheritance, which may help maintain GSC identity and reset chromatin structure in the other daughter cell for proper differentiation (Xie *et al.* 2015).

Utilizing CO-FISH (chromosome orientation fluorescence *in situ* hybridization) probes, which allow strand-specific hybridization of sister chromatids, it has been demonstrated that both X and Y sister chromatids exhibit an ∼85:15 bias during male GSC asymmetric division. Autosomes, specifically chromosome 2 and 3, display a random segregation pattern, but do show a remarkable cosegregation mode (*i.e.*, WW: CC instead of WC: CW. W, Watson strand; C, Crick strand) [Figure 2, (Yadlapalli and Yamashita 2013)]. An earlier study using the nucleoside analog 4-bromo-2-deoxyuridine (BrdU) incorporation assay demonstrated that male GSCs do not follow the immortal strand model (Yadlapalli *et al.* 2011)—a model that hypothesizes that stem cells retain a template copy of DNA, specifically the sister chromatid that contains the oldest strand as a template, to avoid accumulation of DNA replication-induced mutations (Cairns 1975). Since sister chromatids are identical DNA copies of each other, the distribution of distinct information for asymmetric inheritance likely occurs through epigenetic mechanisms, which is consistent with an alternative hypothesis (Klar 2007; Lansdorp 2007).

### Histone-modifying enzymes and factors that affect histone modification(s)

#### Cell autonomous mechanisms:

Recent research has identified a set of specific enzymes and factors that generate (“write”), recognize (“read”), and remove (“erase”) histone modifications, provoking studies of their *in vivo* functions during development (Sarmento *et al.* 2004; Seligson *et al.* 2005). Post-translational modifications that decorate canonical histones (*i.e.*, H2A, H2B, H3, and H4), as well as histone variants, such as H3.3 and H2Av, can serve as molecular memory bookmarks to maintain, or reestablish, transcriptional activation or repression after mitosis. Indeed, different histone modifications are very robust in the male germline (Hennig and Weyrich 2013). The study of histone-modifying enzymes in *Drosophila* offers a great opportunity because many of them encode the sole ortholog, making interpretation of their endogenous roles unambiguous (Table 2).

**Table 2:** PTMs and the corresponding enzymes in *Drosophila* gametogenesis

PTM	Writer	Reader	Eraser	Function	Reference
H3K4(me)	dset1, trr, trx, and ash1	Phf7	lid, Su(var)3-3 (lsd1)	Commonly associated with promoters of actively transcribed gene	Beisel *et al.* (2002), Sedkov *et al.* (2003), Di Stefano *et al.* (2007), Hallson *et al.* (2012), Yang *et al.* (2012), Tarayrah *et al.* (2015)
H3K9(me)	Su(var)3-9, eggless/dSETDb1, dG9a	Rhino (HP1d), HP1a, HP1b, HP1e	dKDM4B	A mark of constitute and facultative heterochromatin, functions in the maintenance of pericentric heterochromatin	Tschiersch *et al.* (1994), Ebert *et al.* (2006), Mis *et al.* (2006), Stabell *et al.* (2006), Levine *et al.* (2015)
H3K23(acetyl)	enok	N.D.	N.D.	Promotes gene expression	Scott *et al.* (2001), Huang *et al.* (2014)
H3K27(me)	E(z)	Pc	dUTX	Associated with inactive gene promoters, indexing both pericentric heterochromatin and inactive euchromatic domains	Min *et al.* (2003), Ebert *et al.* (2004), Tarayrah *et al.* (2013)
H2A (Ub)	dRing	N.D.	PR-DUB (calypso/Asx)	Promotes repressive chromatin	Wang *et al.* (2004), Scheuermann *et al.* (2010)
H2B(Ub)	dBre1	N.D.	Scrawny	Enhances nucleosome stability and mediates H3k4(me)	Buszczak *et al.* (2009), Xuan *et al.* (2013)

##### PcG and TrxG:

Increasing evidence indicates that the Polycomb group (PcG) and the Trithorax group (TrxG) complexes play critical roles for cells to decide between maintaining the proliferating precursor state, and initiating the terminal differentiation program (Ringrose and Paro 2004). It is generally agreed that both PcG and TrxG complexes employ epigenetic mechanisms that alter chromatin state to either repress or activate gene expression (Surface *et al.* 2010). PcG proteins act in at least two distinct, but interacting, protein complexes, Polycomb Repressive Complex 1 (PRC1) and PRC2 (Schwartz and Pirrotta 2007). PRC2 contains an enzymatic component, Enhancer of Zeste [E(z)], which methylates histone H3 at Lys27 (H3K27me3) (Cao *et al.* 2002; Czermin *et al.* 2002; Kuzmichev *et al.* 2002; Muller *et al.* 2002). This methylated histone recruits PRC1, which binds to the H3K27me3 epigenetic mark through the chromodomain of the Polycomb (Pc) protein (Fischle *et al.* 2003; Min *et al.* 2003), leading to the nucleation of the entire PcG complex. It has been shown that mutation on the H3 Lys27 residue phenocopies *loss-of-function* mutants of PRC2 components, indicating that H3K27 is indeed the critical *in vivo* substrate of PRC2 histone methyltransferase activity (Pengelly *et al.* 2013). In addition, the dRing in the PRC1 complex acts as an E3 ubiquitin ligase, which ubiquitylates histone H2A at Lys119 (H2AK119ub) (Wang *et al.* 2004). H2AK119ub may affect transcription by blocking efficient elongation (Stock *et al.* 2007). Phosphorylation of the H2A variant H2Av (γH2Av), which serves as a specific marker for double-strand DNA breaks, often indicates an early response to DNA damage. Consistently, it was reported that mutations in H2Av enhance *Drosophila* male germline defects caused by DNA damage that initially results from mutations in the PcG gene *multi sex combs* (*mxc*) (Landais *et al.* 2014).

On the other hand, the active H3K4me3 mark is generated by the TrxG complex (Byrd and Shearn 2003; Klymenko and Muller 2004; Ringrose and Paro 2004), and opposes the PcG function. In the female GSCs, it has recently been shown that the global level of H3K4me3 is decreased upon loss-of-function of the *Drosophila* ortholog of Ctr9, a component of the Paf1 complex normally required for transcriptional initiation and polyadenylation. However, the functional readout of this global H3K4me3 loss remains unclear (Chaturvedi *et al.* 2016).

In *Drosophila*, not only the maintenance of GSCs, but also the formation of primordial germline cells during embryogenesis, depends on the cell-autonomous function of Piwi (Megosh *et al.* 2006; Ma *et al.* 2014). Piwi promotes GSC differentiation nonautonomously through somatic gonadal cells in both male and female gonads (Ma *et al.* 2014; Gonzalez *et al.* 2015). The necessity of Piwi function for ovarian GSC maintenance, however, does not seem to be relevant to biogenesis of Piwi-interacting RNAs (piRNAs). The Piwi-piRNA complex is required for transposon silencing in the nucleus; however, Piwi in cytoplasm is shown to be sufficient for GSC maintenance (Klenov *et al.* 2011). The 3R-TAS1 piRNA is a specific Piwi-bound piRNA and is involved in female GSC maintenance. However, its role and the mode of action remain elusive (Yin and Lin 2007). Piwi function in GSC maintenance is conserved in other organisms, including mouse and zebrafish, where PIWI-family proteins are also involved in GSC maintenance and/or differentiation (Houwing *et al.* 2007, 2008; Unhavaithaya *et al.* 2009). Recently, it has been demonstrated that Piwi physically interacts with PRC2 components, and restricts the accessibility of PRC2 to chromatin (Peng *et al.* 2016).

##### Eggless:

*eggless*/*dSETDB1* encodes a H3K9 methyltransferase which plays multiple roles in *Drosophila* oogenesis (Clough *et al.* 2014). The *eggless* mutants show female GSC self-renewal defects. However, the GSC loss defect is not caused by loss-of-function in the well-known BMP signaling pathway, suggesting a potential H3K9me3-dependent, but BMP-independent, mechanism for GSC maintenance (Wang *et al.* 2011). Another study showed that *eggless* mutant female flies have a defective egg chamber owing to its requirement for FSC proliferation and survival of both FSCs and germ cells (Clough *et al.* 2007). In addition, it has been shown that Eggless functions in the transcriptional regulation of piRNA clusters (Rangan *et al.* 2011). Activated piRNAs act with the PIWI protein to protect germline genome by preventing transposable element activity, which may be a conserved mechanism for germline genome integrity across species (Malone *et al.* 2009; Sienski *et al.* 2012; Huang *et al.* 2013; Le Thomas *et al.* 2013; Rozhkov *et al.* 2013).

##### Scrawny:

*Scrawny* encodes a deubiquitylating enzyme and targets the mono-ubiquitylation H2B, which normally serves as an active mark for transcription. Therefore, the normal function of Scrawny is to make chromatin more compact to repress gene expression. Interestingly, Scrawny is required for stem cell maintenance in multiple adult stem cell systems, including both female and male GSCs, as well as FSCs in the ovary, and intestinal stem cells, suggesting some common chromatin feature among different stem cell types. It is likely that Scrawny maintains stem cells by repressing transcription of the differentiation genes in the corresponding lineages. In addition, inactivation of Scrawny leads to global changes of the chromatin landscape, including increased levels of H3K4me3 and acetylated H3, suggesting crosstalk among different histone modifications, likely through their modifying enzymes. Another intriguing feature is that Scrawny has highly enriched nucleolar localization in both female and male germline (Buszczak *et al.* 2009). In another study, enhanced ribosomal RNA transcription at nucleolus was shown to maintain normal female GSC proliferation and avoid precocious differentiation (Q. Zhang *et al.* 2014). It is unclear whether Scrawny is required for proper chromatin structure at rDNA genes for their upregulated transcription.

##### Little imaginal disc (Lid):

In adult testis, the H3K4me3-specific histone demethylase Lid has cell-autonomous roles to maintain GSC self-renewal and prevent GSCs from undergoing precocious differentiation. When the function of Lid is compromised in early-stage germ cells, the niche is deprived of GSCs, but occupied by differentiating spermatogonial cysts. The key downstream effector of Lid is the Stat92E transcription factor of the JAK-STAT signaling pathway. Lid is required for both normal Stat92E transcript level and protein accumulation. Removing one copy of Stat92E greatly enhanced *lid* mutant phenotype, and expression of a *Stat92E* cDNA in early-stage germ cells rescued *lid* loss-of-function phenotype completely. Therefore, Lid acts through Stat92E in regulating male GSC activity (Tarayrah *et al.* 2015).

##### Stonewall (Stwl):

*Stwl* encodes a DNA-binding protein, which was originally predicted as a putative transcription factor. More recent work suggests that Stwl normally represses expression of many target genes, likely through making the chromatin structure more compact. Stwl is both necessary and sufficient for female GSC cell fate (Maines *et al.* 2007), as well as the transit amplification of CBs (Akiyama 2002). It has been shown that both H3K9me3 and H3K27me3, two histone modifications to silence gene expression, are decreased in *Stwl* mutants (Yi *et al.* 2009), suggesting that Stonewall maintains normal heterochromatin structure, as one of its functions.

##### longitudinals lacking (lola):

In the male GSC lineage, a transcriptional regulator of the BTB-Zinc finger family encoded by *lola* was reported to have pleiotropic roles in adult testis. Lola is ubiquitously expressed and is required cell-autonomously for both GSC and CySC maintenance, likely independent of the known JAK-STAT and BMP signaling pathways. In addition, *lola* is required for proper mitosis-to-meiosis transition, and *lola* mutant spermatogonial cysts have >16 cells, likely owing to faster cell cycle progression. Finally, *lola* is also needed for meiosis and terminal differentiation of sperm. Intriguingly, *lola* has 19 alternative spicing isoforms. With the possibility that Lola may act as a dimer, the combination among different isoforms could give rise to its pleiotropic roles (Davies *et al.* 2013). By contrast, in female GSCs, *lola* is repressed by Stwl and is dispensable for female GSC maintenance (Maines *et al.* 2007), suggesting sex-specific roles of Lola in gametogenesis.

#### Non-cell-autonomous mechanisms:

A fundamental question in stem cell biology is how extrinsic signaling pathways and intrinsic epigenetic mechanisms cooperate to determine and maintain stem cell fate. Recent findings provide new insights into the non-cell-autonomous roles of different histone-modifying enzymes, many of which are acting through signaling pathways and required for crosstalk among multiple cell types within the stem cell niche.

##### Lysine-specific histone demethylase 1 (Lsd1):

The *lsd1* gene encodes the H3K4me1/2-demethylase in *Drosophila* (Di Stefano *et al.* 2007). Lsd1 acts in escort cells to regulate a diverse group of genes, including both BMP-related and BMP-unrelated genes (Eliazer *et al.* 2014). Lsd1 regulates germline differentiation by preventing ectopic BMP signaling outside of the niche (Eliazer *et al.* 2011, 2014), as well as using BMP-independent mechanisms.

##### Enhancer of zeste [E(z)]:

E(z) is a key PRC2 component, which is an H3K27me3-specific methyltransferase (Muller *et al.* 2002). In *Drosophila* testis, E(z) acts in somatic gonadal cells to prevent expression of a somatic lineage transcription factor encoded by *zinc-finger homeodomain protein 1* (*zfh-1*) in the germline. Consensus holds that germ cells maintain their unique identity after being specified early in embryogenesis, which is essential for proper gametogenesis. Using complementary somatic and germline lineage-tracing experiments, Zfh-1 was shown to ectopically express in the germline in adult testes when *E(z)* is inactivated in the somatic cells, suggesting the importance of cell–cell communication in maintaining germ cell identity. Furthermore, only early-stage germ cells, including GSCs, retain the ability to express *zfh-1*. In contrast, further differentiated spermatogonial cells lose this ability, suggesting that chromatin undergoes structural changes during GSC differentiation that may lock their cell fate choice (Eun *et al.* 2014).

##### Posterior sex combs (Psc) and suppressor of zeste 2 [Su(z)2]:

Both Psc and Su(z)2 are PRC1 components which have some redundant functions. Loss of both genes in ovary leads to overproliferation and “metastasis” of FSCs, likely from misregulation of the canonical Wnt signaling and planar polarity pathways (Li *et al.* 2010). In testis, both Psc and Su(z)2 act in the CySC lineage to maintain their identity and restrict excess proliferation. Loss of both *Psc* and *Su*(*z*)*2* leads to tumors that arise from overproliferative CySCs, which also physically displace GSCs from their niche (Morillo Prado *et al.* 2012).

##### Utx histone demethylase (dUTX):

The *dUTX* gene encodes an H3K27me3-specific histone demethylase (Herz *et al.* 2010). In the testis niche, dUTX removes the repressive H3K27me3 histone modification near the transcription start site of *Socs36E* and allows active transcription of *Socs36E*, which encodes an inhibitor of the JAK-STAT signaling pathway. JAK-STAT plays an essential role in the testis niche; as such, dUTX is critical for maintaining the balance between GSCs and CySCs. Loss of *dUTX* function in either GSCs or CySCs leads to niche cell identity and morphological defects in a non-cell-autonomous manner. These defects can be fully rescued by either overexpression of *Socs36E* or removal of one copy of the downstream transcription factor-encoding gene, *Stat92E*, of the JAK-STAT pathway. Therefore, through direct control of JAK-STAT signaling, dUTX coordinates crosstalk among different cell types within the *Drosophila* testis niche (Tarayrah *et al.* 2013).

##### Enoki mushroom (Enok):

The *enok* gene encodes a putative MYST family histone acetyltransferase that controls female GSC maintenance, both cell-autonomously and non-cell-autonomously (Xin *et al.* 2013). Loss of *enok* in female GSCs leads to rapid GSC loss. Enok maintains GSCs through regulating *Bruno*, which encodes an RNA-binding protein and targets mRNAs in the ovary for translational repression. Furthermore, compromised *enok* in cap cells impairs niche size and BMP signaling output, thereby causing defective GSC maintenance through a parallel non-cell-autonomous pathway.

##### dBre1:

*dBre1* encodes an E3 ubiquitin ligase required for mono-ubiquitination of H2B. The dBre1 controls both GSC maintenance and germ cell differentiation via distinct mechanisms (Xuan *et al.* 2013). Loss of *dBre1* leads to both GSC loss and a significant reduction in H3K4me3. Further analysis revealed that *dBre1* regulates GSC maintenance through modulating BMP signaling response. In addition, dBre1 has a non-cell-autonomous role to maintain GSCs via DE-cadherin-mediated adhesion of GSCs to the niche, as well as the BMP signaling pathway. Finally, dBre1 functions in escort cells to control female germ cell differentiation in a non-cell-autonomous manner through limiting BMP signaling output by downregulating the BMP ligand (Dpp) and Dally—a regulator of BMP ligand diffusion. Interestingly, loss of dSet1—an H3K4 methyltransferase—results in phenotypes similar to those observed in *dBre1* mutant ovaries. Genetic analysis suggests that *dBre1* interacts with *dSet1* to control both female GSC maintenance and germ cell differentiation.

### RNA-binding proteins and noncoding RNAs

RNA-binding proteins, such as Musashi (Msi) (Siddall *et al.* 2006), Held-out-wings (HOW) (Monk *et al.* 2010), and the IGF-II mRNA-binding protein (Imp) (Toledano *et al.* 2012) are all required for male GSC maintenance, suggesting an important role of post-transcriptional regulation in the testis niche.

Epigenetic regulation is also controlled by noncoding RNAs, such as microRNAs (miRNAs) that regulate gene expression post-transcriptionally. Mature miRNAs are ∼22 nt, and they are processed from primary miRNAs by a set of evolutionally conserved enzymes, such as RNase III type endonucleases Partner of drosha (Pasha), Loquacious (Loqs), Dicer-1 (Dcr-1), and Argonaute-1 (Ago-1) (Filipowicz *et al.* 2008) in *Drosophila*. By base-pairing to the 3′ untranslated regions (3′UTR) of target mRNAs (Gu *et al.* 2009), mature miRNAs either control target mRNA stability or interfere with its translation (Vasudevan *et al.* 2007; Filipowicz *et al.* 2008). Mutations of miRNA biogenesis components *dcr-1*, *loquacious*, *argonaut 1*, and *mei-P26* lead to the loss of female GSCs (Forstemann *et al.* 2005; Jin and Xie 2007; Park *et al.* 2007; Yang *et al.* 2007; Li *et al.* 2012, 2013). A couple of miRNAs have also been found to regulate GSC maintenance and differentiation in female gonads. For example, *miR-184* (Iovino *et al.* 2009) and *bantam* (Yang *et al.* 2009) are both required to balance GSC maintenance *vs.* differentiation in ovaries. Another example is male germline-specific regulation of the Wnt signaling pathway by miRNAs. Both β-catenin and the downstream transcription factor TCF are downregulated by miRNAs in *Drosophila*. Loss of this antagonization leads to male germline differentiation defects and decreased fertility (Pancratov *et al.* 2013).

Another class of small noncoding RNAs, called piRNAs, is present in the both male and female gonads. The piRNAs are the most abundant class of small RNAs in gonads. The piRNAs repress transposons, provide immunity against transposons to protect the next generation, and function in maternal-to-zygotic transmission during early embryogenesis (Brennecke *et al.* 2008; Barckmann *et al.* 2015; Hermant *et al.* 2015; Iwasaki *et al.* 2015; Vourekas *et al.* 2016). Their biogenesis and functions will be described in detail in the following sections.

## Mechanisms Controlling Mitotic Germ Cell Proliferation, Transition to Meiosis, and Dedifferentiation

### Transit-amplification stage and mitosis-to-meiosis transition

The transit-amplification stage ensures that limited GSCs and their divisions have a high-throughput outcome for producing gametes. However, in both female and male germlines, this process needs to be tightly controlled since genetic lesions or epigenetic misregulation of gene expression may trap them as ever-dividing mitotic cells, and block entry into meiosis, leading, in turn, to either germline tumors or infertility (Clarke and Fuller 2006).

#### Cell-autonomous mechanisms:

A key differentiation factor encoded by the *bam* gene is expressed in transit-amplifying cells in both female and male gonads (McKearin and Spradling 1990; Gonczy *et al.* 1997). In GSCs from both sexes, BMP signaling activated by somatic cells in the niche represses *bam* transcription (Shivdasani and Ingham 2003; Kawase *et al.* 2004; Schulz *et al.* 2004). It is important that *bam* remain silenced in GSCs (Schulz *et al.* 2004; Insco *et al.* 2009; Monk *et al.* 2010). Ectopic expression of *bam* in GSCs induces precocious differentiation or cell death and, hence, loss of GSC phenotype (Ohlstein and McKearin 1997; Schulz *et al.* 2004; Sheng *et al.* 2009). A recent study shed light on the biochemical activity of Bam protein by showing that it assists a deubiquitinating enzyme and protects CycA from degradation (Ji *et al.* 2017).

In the female GSC lineage, Bam is absent in the GSC, but it is expressed in its immediate daughter cell CB. This abrupt change of Bam expression is regulated by a steep gradient of response to BMP signaling, both by a *cis*-acting transcriptional silencer repressing *bam* transcription in GSCs (Chen and McKearin 2003a,b; X. Song *et al.* 2004) and by a *trans* acting mechanism, such as the CB-specific degradation of Thickveins—a BMP signaling receptor (Xia *et al.* 2010, 2012). In addition, post-transcriptional regulation via different RNA-binding proteins contributes to sharpening the change of Bam expression and the decision between GSC self-renewal and CB differentiation (Chen and McKearin 2005). Further differentiation of the CB relies on the homolog of human Ataxin 2-Binding Protein 1 (A2BP1), which is expressed immediately after Bam in 4- to 16-cell germline cysts. Mutations in the *A2BP1* gene lead to germline cyst differentiation defects, giving rise to germline tumors that result from mitosis-to-meiosis transition defects (Tastan *et al.* 2010). In addition, two H3K9 methyltransferases encoded by *eggless*/*dSETDB1* and Su(var)3-9 act in a sequential manner with dSETDB1 in GSCs and early-stage germline, as discussed previously, while Su(var)3-9 mainly works in germ cells at the later stage. This temporal difference could underlie their distinct loss-of-function phenotypes with *eggless* mutants showing severe germline differentiation defects, whereas *Su*(*var*)*3-9* mutants undergo normal oogenesis (Yoon *et al.* 2008).

In the male GSC lineage, Bam is required for the transition from mitotic spermatogonia to meiotic spermatocytes (McKearin and Spradling 1990; Gonczy *et al.* 1997). Bam protein is detectable in four- to 16-cell spermatogonia with a peak level in eight-cell spermatogonia (Gonczy *et al.* 1997). Examples of post-transcriptional regulation of *bam* include the HOW RNA-binding protein (Monk *et al.* 2010) and *miR-7*, both of which have been implicated in binding to *bam* mRNA and downregulating *bam* expression (Pek *et al.* 2009). Another RNA binding protein, Maelstrom (Mael), is required in spermatogonia to repress *miR-7* and upregulate *bam* expression so that the transit-amplification can proceed normally (Pek *et al.* 2009). The transition from mitotic spermatogonia to meiotic spermatocyte is regulated by the accumulation of Bam to a threshold level. Expediting Bam accumulation or slowing down the transit-amplifying cell cycle results in insufficient proliferation before the transition to meiosis, as demonstrated by spermatocyte cysts with <16 cells. On the other hand, inhibition of Bam accumulation, or facilitating the transit-amplifying cell cycle, results in extra round(s) of mitosis before the transition to meiosis, as shown in spermatocyte cysts with >16 cells (Insco *et al.* 2009). Therefore, expression of Bam needs to be tightly controlled during the transit-amplification stage in the male germline.

Another differentiation gene, *benign gonial cell neoplasm* (*bgcn*), has mutant phenotypes similar to *bam* in both male and female germlines (Gonczy *et al.* 1997). Studies in the female germline demonstrate that Bam and Bgcn form a protein complex to antagonize GSC self-renewal factors, and promote differentiation gene expression in transit-amplifying cells (Y. Li *et al.* 2009). It has been demonstrated that the Trim-NHL tumor suppressor homolog Mei-P26 has a reciprocal regulation with Bam whereby Mei-P26 initially promotes Bam protein accumulation in early transit-amplifying cells. Increased Bam acts with Bgcn to bind the 3′UTR of *Mei-P26*, and, consequently, repress translation of *Mei-P26* in late transit-amplifying cells (Insco *et al.* 2012). Recent studies have revealed another RNA-binding protein, Tumorous testis (Tut), that acts in synergy with Bam-Bgcn for the translational repression of *Mei-P26* (Chen *et al.* 2014). Indeed, post-transcriptional regulation of gene expression is a widely used mechanism, particularly in the germline. A very recent study reports the generality of this mechanism in the male germline (Shan *et al.* 2017). In addition, post-translational regulation also directly, or indirectly, regulates Bam protein function. Specifically, a *Drosophila* homolog of the highly conserved LAMMER/Cdc2-like kinase (CLK), called Doa, has been shown to regulate the mitosis-to-meiosis switch in the male germline through regulating Bam protein (Zhao *et al.* 2013).

In order to study the transcriptional profile and chromatin state in transit-amplifying cells, *bam* or *bgcn* mutant testes were used for transcriptome profiling because they are enriched with overproliferative spermatogonial cells (Terry *et al.* 2006; Gan *et al.* 2010a; Chen *et al.* 2011). High-throughput mRNA sequencing (RNA-seq) studies reveal that both chromatin remodeling factors and histone-modifying enzymes have enriched transcription in *bam* testes compared to wild-type testes (Gan *et al.* 2010a). Furthermore, ChIP followed by high-throughput sequencing (ChIP-seq), revealed a distinct chromatin structure in *bam* testes (Gan *et al.* 2010b). In mouse embryonic stem cells, differentiation genes have both repressive H3K27me3 and active H3K4me3 modifications (*i.e.*, “bivalent” chromatin signature), as well as stalled RNA polymerase II (Pol II, *i.e.*, “poised” genes), at their promoter regions (Bernstein *et al.* 2006; Buszczak and Spradling 2006; Guenther *et al.* 2007). By contrast, differentiation genes required for spermatocyte maturation and spermiogenesis are either enriched with H3K27me3 only, or deprived of both H3K4me3 and H3K27me3, in *bam* testes, and they are not associated with stalled Pol II (Gan *et al.* 2010b). This distinct chromatin structure may prevent ectopic transcription of the differentiation genes in transit-amplifying cells. On the other hand, it suggests that dramatic changes at the promoter region of differentiation genes are needed to turn on their robust transcription in spermatocytes.

In addition to these genome-wide studies, it was reported that an epigenetic reader-encoding *Plant Homeodomain Finger* 7 (*PHF7*) gene is specifically expressed in GSCs and transit-amplifying cells. PHF7 recognizes active H3K4me2 histone modification and is required for GSC maintenance and proper spermatogonial differentiation (Yang *et al.* 2012). Further studies to identity the target genes of PHF7, which should be enriched with H3K4me2 or H3K4me3, will shed light on its *in vivo* roles.

#### Non-cell-autonomous mechanisms:

The Epidermal growth factor (Egf) signaling pathway plays an important role in the regulation of the mitosis-to-meiosis switch. The Egfr (Egf receptor) ligand Spitz is processed by Stet—a transmembrane protease—in germ cells (Schulz *et al.* 2002). Activated Spitz then acts on Egfr expressed in somatic cells (Kiger *et al.* 2000). Egf signaling acts through the guanine nucleotide exchange factor (GEF) Vav to activate Rac-type small GTPases, which are antagonized by the Rho-type small GTPases (Sarkar *et al.* 2007). Egfr signaling acts in cyst cells to restrict GSC self-renewal and spermatogonial proliferation, while promoting GSC-to-GB and spermatogonia-to-spermatocyte transitions (Kiger *et al.* 2000). Egfr signaling decreases the frequency of GSC divisions in the adult, but not larval, testes, suggesting a temporal mode of Egfr regulation (Parrott *et al.* 2012). In addition, mutations in a serine/threonine kinase signal transducer encoded by *raf* result in phenotypes similar to the *Egfr* mutant, suggesting that the receptor tyrosine kinase (RTK) pathway is, in general, required in cyst cells for proper transit-amplification (Tran *et al.* 2000). The direct target genes for the Egfr/Raf pathway have not been identified; however, because compromised Egf signaling leads to defects in germline-soma interaction and overproliferation of spermatogonial cells, it is possible that the target genes regulate proper encapsulation of germ cells by cyst cells (Schulz *et al.* 2002; Sarkar *et al.* 2007). A recent study has shown that a chromatin factor encoded by the *Enhancer of Polycomb* [*E*(*Pc*)] gene acts in the CySC lineage to regulate multiple signaling pathways, including both EGF and JAK-STAT pathways, in order to promote both CySC and GSC differentiation. In addition, consistent with biochemical data showing E(Pc) as a component of the NuA4 (nucleosome acetyltransferase of H4) histone acetyltransferase (HAT) complex (Galarneau *et al.* 2000; Boudreault *et al.* 2003; Chittuluru *et al.* 2011), inactivation of the *Drosophila* NuA4 homolog, Tip60, in the CySC lineage resembles *E*(*Pc*) loss-of-function phenotype, suggesting that they may act together *in vivo* (Feng *et al.* 2017). Another recent study revealed that the endocytic process in the CySC lineage is required to prevent overproliferation of transit-amplifying germ cells in testis, which is accomplished through both JNK and BMP signaling pathways (Tang *et al.* 2017).

Furthermore, a nuclear envelope component, Nucleoporin98-86, regulates proper GSC-to-GB and spermatogonia-to-spermatocyte transitions, and functions upstream of BMP, JAK-STAT, and Egfr signaling pathways (Parrott *et al.* 2011). Interestingly, another study showed that nuclear lamina regulates specific nucleoporin distributions and promotes nuclear localization of phosphorylated ERK—the downstream effector of the Egf pathway (Chen *et al.* 2013). These results highlight the importance of nuclear structure in regulating cellular differentiation during spermatogenesis.

### Dedifferentiation

#### Cell-autonomous mechanisms:

In both female and male GSC lineages, partially differentiated mitotic germ cells could undergo a dedifferentiation process to return to the niche and become GSC-like cells (Brawley and Matunis 2004; Kai and Spradling 2004). During aging (Wallenfang *et al.* 2006; Cheng *et al.* 2008) and tissue regeneration (Sheng *et al.* 2009), lost GSCs could be replenished by dedifferentiation to maintain tissue homeostasis. However, once the meiotic program is initiated, as in spermatocytes, dedifferentiation could no longer be detected (Brawley and Matunis 2004; Wallenfang *et al.* 2006; Sheng *et al.* 2009), suggesting that the mitotic spermatogonial cells have unique characteristics permissive for dedifferentiation. Similar irreversible commitment may also apply to female meiotic germ cells because only four- to eight-cell transit-amplifying cells have been reported to undergo dedifferentiation in the ovary (Kai and Spradling 2004). It has recently been shown that RNA-binding Fox 1 (Rbfox1) represses *pumilio* mRNA translation. Because of the essential roles of Pumilio in early-stage germ cells, including GSCs, ectopic Pumilio is expressed in *Rbfox1* mutants, and promotes dedifferentiation of germline cysts to become GSC-like cells in the ovary (Carreira-Rosario *et al.* 2016).

#### Non-cell-autonomous mechanisms:

Using live cell imaging, it has been observed that the dedifferentiated spermatogonial cyst undergoes fragmentation to become individual cells that form actin-based protrusions to make initial contact with the stem cell niche (Sheng *et al.* 2009), suggesting potential extrinsic cues from the niche to guide dedifferentiation. Indeed, it has been reported that the aminopeptidase Slamdance is highly expressed in the hub cells. Slamdance is both necessary and sufficient to promote dedifferentiation during homeostasis and regeneration, and such activity depends on its enzymatic function. These data showed that cells and molecules in the stem cell niche regulate the dedifferentiation process. Slamdance also has a cell-type-specific expression in the female GSC niche cells, and may play a similar role for the dedifferentiation process in the female germline (Lim *et al.* 2015).

Despite increasing knowledge about the intrinsic factors and extrinsic cues for dedifferentiation, the extent to which dedifferentiated GSC-like cells behave like *bona fide* GSCs remains to be elucidated. For example, it has been shown that dedifferentiated GSC-like cells tend to have misoriented centrosomes, which lead to cell cycle arrest because of a centrosome orientation checkpoint in male GSCs (Cheng *et al.* 2008; Inaba *et al.* 2010, 2015; Yuan *et al.* 2012). Therefore, it is unclear whether dedifferentiated GSC-like cells could reenter differentiation and give rise to fully functional gametes without any defects. Addressing this intriguing question would need double lineage tracing to trace those GSC-like cells arising from dedifferentiation and reentering differentiation, as well as functional analyses of those differentiated gametes from dedifferentiated GSC-like cells.

## piRNAs and piRNA Pathway Function to Protect the Germline Genome

Transposons are autonomous elements present in all eukaryotic organisms. Their content in the genome of higher eukaryotes varies between 10 and 80%; they constitute 23% of the *D. melanogaster* genome. Transposons are involved in the regulation of gene expression, as well as both evolution and speciation. However, the ability of transposons to transpose from one site to another in the genome demands tight regulation of their movements. Transposition in the germline genome is particularly important for transposons to propagate in a population. Previously, piRNAs present in both the male and female gonads were introduced as another class of small noncoding RNAs. It is this unique class of small RNAs that carefully safeguard the germline genome, ensuring fitness of the offspring. Genome-wide screens in *Drosophila* have revealed that piRNA biogenesis requires 69 or more genes and that their distinct subsets are expressed in the germline and somatic gonadal cells (Czech *et al.* 2013; Handler *et al.* 2013). In this section, we focus on the biogenesis of piRNAs in germ cells, as well as somatic gonadal cells.

### Transposons in *Drosophila*

*D. melanogaster* possesses >49 families of long terminal repeat (LTR) transposons (Kaminker *et al.* 2002). The transposition mechanisms for many transposon families have been studied. The most studied example among the LTR family members is *gypsy*, which is composed of three parts: a Gag-like protein containing a nucleocapsid region, a protease-polymerase fusion protein, and the envelope (Mejlumian *et al.* 2002). With the encoded coat proteins, *gypsy* is capable of exiting the follicle cells and infecting the neighboring oocyte. Another LTR retrotransposon called *ZAM* has a replicative cycle similar to that of *gypsy*, but it can be transmitted to the oocyte by the vitellogenin secretion pathway (Leblanc *et al.* 2000; Brasset *et al.* 2006). LTR families are often activated in ovarian somatic cells and transmitted to the oocyte, threatening the genome stability of the oocyte. By contrast, non-LTR families tend to be activated in nurse cells, and are deposited to the oocyte via the cytoplasmic bridges called ring canals (Chambeyron *et al.* 2008). Hence, active transposon mechanisms function both in the germline and somatic gonadal cells.

In *Drosophila*, though transposons threaten the genome, they are also essential for the integrity of both centromeres and telomeres (Pardue and DeBaryshe 2003; Wong and Choo 2004). In most other species, telomeres are composed of simple repeats and are maintained by telomerase, but, in *Drosophila*, telomeres consist of three non-LTR transposons, namely *HeT-A*, *TART*, and *TAHRE* (Pardue and DeBaryshe 2003, 2011; Abad *et al.* 2004). To properly maintain the telomere, the copy number of these transposons at telomeres is strictly regulated (Fanti *et al.* 1998; Perrini *et al.* 2004; Frydrychova *et al.* 2008; Pardue and DeBaryshe 2011).

### piRNAs and PIWI proteins

Studies on the piRNA pathway in *Drosophila* ovaries have expanded our knowledge about piRNA pathway function (Iwasaki *et al.* 2015). The piRNAs in *Drosophila* are 23 to 29 nucleotides in length and are the most abundant small RNAs in gonads (Balakireva *et al.* 1992; Aravin *et al.* 2001, 2003, 2004). The piRNAs were first recognized for their role in suppressing the Stellate protein in the male germ cells (Aravin *et al.* 2001, 2004). Shortly thereafter, it was reported that *Drosophila* ovaries and embryos contain abundant repeat-associated small RNAs called rasiRNAs (Vagin *et al.* 2006). Later, they were renamed as piRNAs, as they are produced by and associate with PIWI-family proteins to suppress transposons (Saito *et al.* 2006; Brennecke *et al.* 2007; Gunawardane *et al.* 2007).

The piRNA pathway in *Drosophila* is active in both the germline and somatic gonadal cells to counter transposons that threaten to invade the germline through distinct routes. The piRNA pathway silences transposons post-transcriptionally by triggering degradation of their transcripts, as well as transcriptionally by silencing transposon loci (Vagin *et al.* 2006; Lim *et al.* 2009; Le Thomas *et al.* 2013; Rozhkov *et al.* 2013). Although a different subset of proteins function in piRNA biogenesis in germline and somatic cells, PIWI-family proteins—a subclade of the Argonaute family—are central in the piRNA pathway. Piwi, the founder member of PIWI-family proteins, is present in both germline and somatic cells. Two other PIWI-family proteins, namely Aubergine (Aub) and Argonaute3 (Ago3), are required for piRNA production in germ cells. These proteins contain PAZ (Piwi-Argonaute-Zwille) and PIWI domains. The PAZ domain also harbors an oligonucleotide/oligosaccharide-binding-fold, which binds to single-stranded nucleic acids (Lingel *et al.* 2003; Yan *et al.* 2003). The PIWI domain is structurally similar to that of RNase H enzymes (J. Song *et al.* 2004). Crystal structure analysis suggested that the PAZ domain forms a pocket for the target RNA, while the PIWI domain cleaves its bound RNA (Yan *et al.* 2003; J. Song *et al.* 2004).

The piRNAs are processed from longer precursor molecules. The piRNA precursor transcripts are produced from discrete loci, termed piRNA clusters (Brennecke *et al.* 2007). These clusters are composed of fragmented copies of transposons in the genome, and serve as heritable sequence repositories for transposon repression. In the gonadal somatic cells, piRNAs are processed from piRNA cluster transcripts, which are in antisense orientation, to active transposons, in a linear mode called primary processing. While in germ cells, piRNAs are generated from both cluster and transposon transcripts in two different modes: primary processing and secondary amplification cycle (Brennecke *et al.* 2007; C. Li *et al.* 2009; Malone *et al.* 2009). The piRNAs in both cell types are loaded onto Piwi to form the Piwi-piRNA complex, which subsequently translocates into the nucleus for transcriptional silencing of transposons.

### Source of piRNAs—piRNA clusters and transcription of piRNA precursors

Generally, piRNA clusters can be classified on the basis of transcription of precursors. Most piRNA clusters that participate in piRNA biogenesis in ovarian somatic cells are transcribed in one direction, and thus called uni-strand clusters. By contrast, active clusters in germ cells are transcribed in a convergent manner from both directions, and thus called dual-strand clusters (reviewed by Hirakata and Siomi 2016).

#### Active piRNA clusters in germ cells and generation of piRNA precursors:

**Figure 3:**
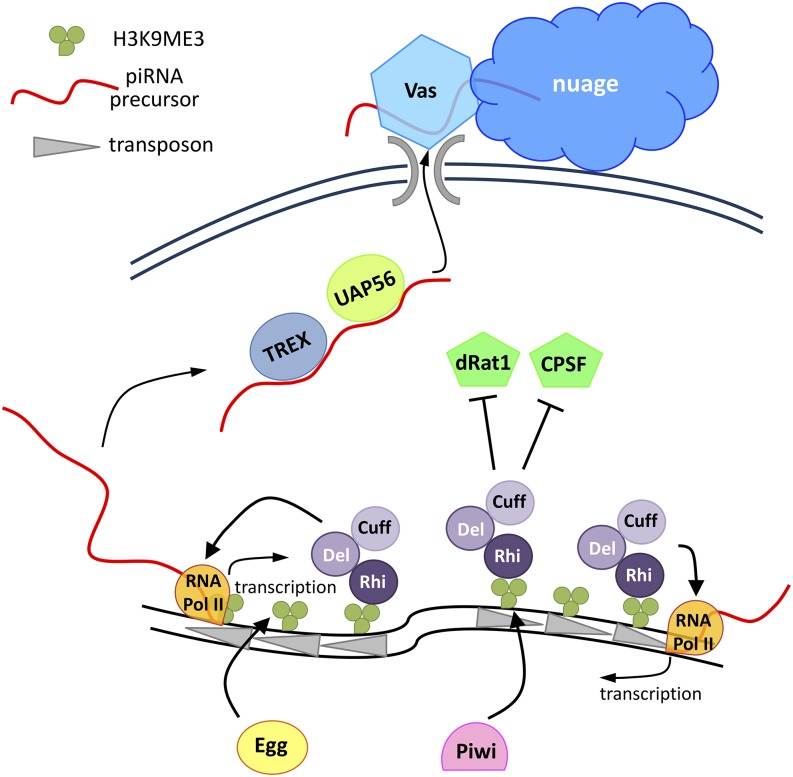
Transcription of bidirectional clusters in the germline. The H3K9me3 marks are deposited on the piRNA clusters by the histone methyl transferase, Egg, and also by Piwi at selected clusters. Rhino recognizes the H3K9me3 marks and prompts binding of the Rhi-Del-Cuff complex (RDC) to the piRNA cluster. The RDC licenses noncanonical transcription from piRNA clusters by recruiting RNA-pol II for transcription of piRNA clusters. Longer transcripts in the antisense direction to transposons are transcribed. Cuff represses Cleavage and Polyadenylation Specificity Factor (CPSF), preventing the termination of cluster transcription, as well as its splicing. In addition, Cuff recruits components of Transcription Export (TREX) complex to stabilize and accumulate cluster transcripts. The Rhi partner, UAP56, binds to the cluster transcripts in order to export them to nuage.

Most clusters active in germ cells are located in pericentric or subtelomeric regions, which are heterochromatic in nature, and yet they are readily transcribed and processed into piRNAs (Klattenhoff *et al.* 2009; Malone *et al.* 2009; Mohn *et al.* 2014; Z. Zhang *et al.* 2014). Although piRNA clusters are transcribed by RNA Pol II, majority of piRNA precursors are reported as nonpolyadenylated and lack capping at the 5′-end (Mohn *et al.* 2014; Z. Zhang *et al.* 2014; Chen *et al.* 2016) (Figure 3). Notably, piRNA precursor transcription depends on H3K9me3, and loss of a histone methyltransferase encoded by *setDB1/egg* causes severe reduction in cluster transcript levels from both uni- and bidirectional clusters (Rangan *et al.* 2011). A recent study showed that loss of *rpp30*, which encodes a subunit of RNase P for tRNA processing, leads to reduction of H3K9me3 at piRNA clusters and reduction in levels of cluster transcripts, supporting the importance of H3K9me3 for cluster transcription (Molla-Herman *et al.* 2015).

Transcription from piRNA clusters requires specialized complexes because many such clusters are present in gene-poor regions and lack canonical promoters, as well as canonical intron-exon boundaries. In the germline, the RDC (Rhino-Deadlock-Cutoff) protein complex licenses transcription from piRNA clusters (Mohn *et al.* 2014; Z. Zhang *et al.* 2014) (Figure 3). RDC is composed of Rhino (Rhi), a homolog of Heterochromatin Protein 1a (HP1a), Deadlock (Del) without any conserved domain, and Cutoff (Cuff), an Rai1/Dom3Z-family protein (Chen *et al.* 2007; Klattenhoff *et al.* 2009; Mohn *et al.* 2014; Z. Zhang *et al.* 2014). The chromodomain on Rhino recognizes H3K9me3 marks at clusters for the binding of RDC complex (Mohn *et al.* 2014; Z. Zhang *et al.* 2014; B. Yu *et al.* 2015). However, the detailed mechanism underlying the establishment of H3K9me3 at clusters is not known. The RDC complex binds to all dual-strand piRNA clusters, which are active in the germline, but not to the clusters active in somatic cells. The RDC complex prevents transcription termination of adjacent genes to allow for transcription of the clusters. The RDC complex also licenses transcription from noncanonical promoters in piRNA clusters (Le Thomas *et al.* 2014; Mohn *et al.* 2014; Z. Zhang *et al.* 2014). Loss of RDC complex leads to an increase in splicing of cluster transcripts, which could potentially destabilize these cluster transcripts (Mohn *et al.* 2014; Z. Zhang *et al.* 2014; Chen *et al.* 2016). Indeed, it was shown that RDC tethering to a transgene leads to intron stabilization and transcription beyond the polyA site. Aravin and colleagues suggest that Cuff in the RDC complex is necessary to prevent binding of Cleavage and Polyadenylation Specific Factor (CPSF) for a continuous transcription throughout the piRNA cluster (Chen *et al.* 2016). Cuff is also required for stabilizing the noncapped cluster transcripts, by antagonizing cluster transcript destabilization by 5′-3′ exonuclease dRat1 (Chen *et al.* 2016). The RDC complex is important not only for transcription from the piRNA clusters, but also for transcription and piRNA production from transgenes (Z. Zhang *et al.* 2014). In addition, RDC complex also participates in channeling the cluster transcripts to the piRNA processing site for piRNA generation.

Recently, it was reported that Tho5 and other THO subunits of the Transcription/Export (TREX) complex are recruited to piRNA clusters by Cuff, and are loaded onto cluster transcripts (Hur *et al.* 2016) (Figure 3). Tho proteins are required for accumulation of nascent cluster transcripts in a splicing-independent manner in nucleus. Another protein, UAP56, a component of the nuclear pore complex, binds to cluster transcripts in a Rhi-dependent manner. Through interaction with a nuage component, Vasa, an RNA helicase, UAP56 likely functions to export cluster transcripts to nuage, the site of piRNA processing (Zhang *et al.* 2012) (see below).

#### Transcription of piRNA clusters active in the ovarian somatic cells:

Somatic piRNA clusters do not require the RDC complex for their transcription. Details about their transcription remain limited. In contrast to germline cluster transcripts, somatic piRNA cluster transcripts are polyadenylated. A piRNA cluster, *flamenco*, located near the pericentric region of X-chromosome, is particularly active in the ovarian follicle cells. The *flamenco* locus has fragmented copies of transposons expressed in somatic cells, such as *ZAM* and *gypsy* (Prud’homme *et al.* 1995; Sarot *et al.* 2004; Desset *et al.* 2008). The *flamenco*-derived transcripts are in antisense orientation to the active transposons. Transcription from the *flamenco* locus is reported to be dependent on the transcription factor Cubitus interruptus (Ci). The *flamenco*-derived transcripts are alternatively spliced, probably for diversity (Goriaux *et al.* 2014).

### piRNA biogenesis in somatic gonadal cells via primary processing

The somatic piRNA cluster transcripts are processed into piRNAs in the cytoplasm (Haase *et al.* 2010; Saito *et al.* 2010). Mechanisms of transport of somatic piRNA precursors from the nucleus to cytoplasmic processing sites remain elusive. Most primary piRNA processing components are localized to mitochondria. Yb-body and Zucchini (Zuc)-associated proteins have been shown to coordinate piRNA processing and loading of piRNAs to Piwi (Haase *et al.* 2010; Saito *et al.* 2010; Handler *et al.* 2011; Qi *et al.* 2011). The 5′-end of piRNAs is generated by an exonuclease, Zucchini (Zuc), which localizes to the mitochondrial surface (Ipsaro *et al.* 2012; Nishimasu *et al.* 2012). Zuc generates 5′-ends of piRNAs, the resultant ends of which are not enriched with U (Uracil). However, the 5′-ends of mature piRNAs are remarkably biased for U. *In vitro* study of the silkworm Piwi homolog Siwi suggested that this bias for U at the 5′-end could be introduced during piRNA loading to Siwi (Kawaoka *et al.* 2011). The 3′-ends of piRNAs are speculated to be generated while piRNAs are being loaded to PIWI proteins. Zuc is thought to function in 3′-end formation of piRNAs, although the resultant products have additional nucleotides at the 3′-end. These extra nucleotides are proposed to be trimmed by another exonuclease (Kawaoka *et al.* 2011). A Tudor-domain protein, PAPI, has also been suggested to be important for 3′-end trimming of piRNAs, although PAPI does not have any nuclease domain (Honda *et al.* 2013). The piRNAs are loaded to Piwi at Yb-bodies present at the outer mitochondrial surface close to Zuc (Olivieri *et al.* 2010; Saito *et al.* 2010). Piwi forms a complex with Yb-body proteins, such as Vretreno (Vret), Shutdown (Shu), and Armitage (Armi). Severe reduction of piRNAs in *vret*, *shu*, and *armi* mutants leads to cytoplasmic accumulation of Piwi, which likely results from the failure of piRNA loading on Piwi (Haase *et al.* 2010; Olivieri *et al.* 2010, 2012; Saito *et al.* 2010; Handler *et al.* 2011; Qi *et al.* 2011). In addition, Shu functions for piRNA loading onto Piwi with the help of Hsp 83 (Handler *et al.* 2011).

The Yb-body, where many proteins required for primary piRNA processing are localized, is named after the Yb protein containing both an RNA-helicase and a Tudor domain (King *et al.* 2001; Szakmary *et al.* 2009). Yb proteins bind to the *flamenco*-derived piRNA precursors through their DEAD box domain, channel them to the Yb-body, and stabilize the piRNA processing apparatus at Yb-bodies (Murota *et al.* 2014). Although all Yb-body components are required for piRNA biogenesis, their exact function remains unknown.

### piRNA biogenesis in germ cells

In addition to primary piRNA processing, germ cells have an additional piRNA processing machinery called secondary amplification or the ping-pong cycle. This piRNA biogenesis mechanism allows more robust piRNA production against transposons and provides more flexibility to adapt for newer transposon threats.

#### Primary piRNA biogenesis in germ cells:

The mechanistic details of primary piRNA biogenesis in germ cells remain unknown. However, many components required for primary piRNA processing in ovarian somatic cells, such as Zuc, Armi, Gasz, Shu, Mino, and HSP90, are expressed in germ cells and suggested to function for primary piRNA processing in germ cells.

#### Secondary piRNA processing in germ cells; ping-pong amplification:

Secondary piRNA processing is a feed-forward amplification loop involving two PIWI-family proteins, Aub and Ago3, and it takes place at the nuage in germ cells where the key components of this secondary processing are localized. The Aub-bound antisense piRNAs target the transposon transcripts, and piRNA-loaded Aub harboring slicer activity cleaves the transposon transcript, generating the 5′-end of transposon-derived sense piRNA. The 3′-end of the piRNAs is generated either by slicer activity or by Zucchini. Ultimately the 3′ end generated by both the mechanisms requires trimming by an exonuclease Nibbler, generating mature Ago3-bound sense piRNAs of correct size. (Nishimasu *et al.* 2012; Hayashi *et al.* 2016). In turn, piRNA-bound Ago3 targets and cleaves cluster transcripts to generate more antisense piRNAs loaded onto Aub or Piwi, and the processing cycle amplifies piRNAs in a feed-forward loop. This processing leads to a significant 10-nt overlap between Aub and Ago3-bound piRNA sequences, with a U at position 1 of Aub-bound piRNAs and an adenine (A) at position 10 of Ago3-bound piRNAs (Brennecke *et al.* 2007; Gunawardane *et al.* 2007). Computational analysis suggested that Aub preferentially binds to mRNA targets with an A at the position opposing the first base of their piRNA partner. Upon target slicing and subsequent piRNA maturation, the A of target mRNA comes to the 10th position in a ping-pong-derived piRNA (Wang *et al.* 2014).

**Figure 4:**
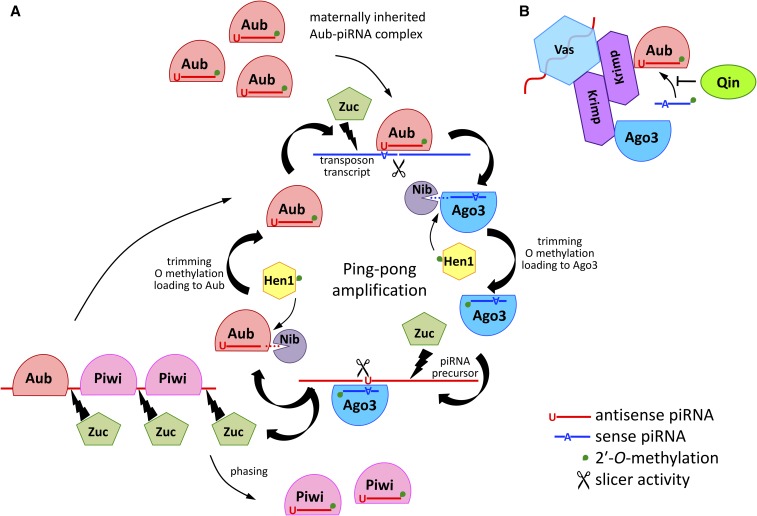
piRNA biogenesis mechanisms in germ cells. (A) The piRNA production mechanisms at nuage. (i) Ping-pong amplification: Aub-bound antisense piRNA leads to the cleavage of the transposon transcript. The 5′-end of piRNAs is produced by Zucchini, while the 3′-end is either generated by Zucchini or by a splicer complex. The resulting 3′ end is trimmed by an exoribonuclease Nibbler, and subsequently, is methylated at 2′-O. The piRNA-Ago3 complex cleaves the anti-sense piRNA precursor transcript. Trimming of the resultant piRNAs occurs as described above. This creates a feed-forward amplification loop, called the ping-pong cycle. (ii) Phasing: The Ago3 cleaved transcript at 5′ end, bound by Aub, apart from going to ping-pong amplification, enters phasing. Piwi is proposed to bind downstream of the Aub cleavage site on a transposon transcript. Zuc cleaves at 5′- and 3′-ends of the transcript bound by Piwi. The Piwi-bound piRNAs, thus resulting from Zuc mediated cleavage, have their 5′-end 27 nucleotides apart from each other, leading to production of piRNAs in a phased manner. This phasing produces piRNAs beyond the Aub and Ago3 cleavage sites and broadens the piRNA repertoire to target transposon threats. (B) Tudor domain proteins, Krimp and Qin, ensure that the ping-pong cycle occurs at the nuage. Vas receives the transcripts of piRNA clusters at nuage. Krimp anchors both Aub and Ago3 at nuage, while Qin inhibits the loading of sense piRNAs on Aub, thus enforcing heterotypic ping-pong.

The secondary piRNAs also trigger production of Zuc-dependent, 3′-directed phased piRNAs. Phasing is triggered by so-called responder piRNAs, which result from Ago3-piRISC activity in the ping-pong cycle. The piRNAs downstream of responder piRNAs are associated with Piwi. These piRNAs are designated as trailer piRNAs. The responder piRNAs and trailer piRNAs show marked phasing with a ∼27 nt interval and a striking bias for U at the 5′-end. Production of these trailer piRNAs depends on Zuc. These findings also suggest that piRNA 3′-ends are defined by Zuc endonucleolytic activity (Han *et al.* 2015; Mohn *et al.* 2015) (Figure 4). Biogenesis of such phased piRNAs spreads piRNA production beyond the target cleavage sites of Ago3 and Aub, thus allowing sequence diversification in the piRNA pool, which could target transposon threat in an adaptive manner by both TGS (Transcriptional Gene Silencing) and PTGS (Post-Transcriptional Gene Silencing) mechanisms (Han *et al.* 2015; Homolka *et al.* 2015; Mohn *et al.* 2015; Sato *et al.* 2015; Senti *et al.* 2015; Wang *et al.* 2015; Webster *et al.* 2015).

#### Nuage as a site for ping-pong cycle in germ cells:

Both PIWI-family proteins, Aub and Ago3, involved in ping-pong amplification are found at the perinuclear foci in the cytoplasmic face called nuage (Brennecke *et al.* 2007). Nuage is an amorphous, electro-dense structure present at the cytoplasmic face of nuclear membrane (reviewed in Eddy 1975). Nuage has been widely recognized, albeit occasionally by different names, as a hallmark of germ cells in animals, and yet its function remained unknown for a long time.

Studies have shown that different kinds of proteins localize to nuage and participate in piRNA processing, including RNA helicase Vasa and Spindle-E (SpnE). Most nuage components are Tudor domain proteins, such as Tudor (Tud), Qin/Kumo, Tejas (Tej), Tapas (Tap), Krimp (Krimp), and SpnE. Other proteins include HMG box protein Mael and nucleases, such as Zuc and Squash (Squ) (Lim and Kai 2007; Pane *et al.* 2007; Malone *et al.* 2009; Patil and Kai 2010; Zhang *et al.* 2011; Anand and Kai 2012; Sienski *et al.* 2012; Patil *et al.* 2014). Tudor domains preferably bind to symmetrical demethylation of Arginine (sDMA) sites on PIWI-family proteins (Nishida *et al.* 2009). Tud binds to Aub and Ago3 in an sDMA-dependent manner and ensures proper binding of piRNAs to Aub and Ago3 (Nishida *et al.* 2009). However, the interaction between Tudor domains and PIWI-family proteins may not always be dependent on sDMA (Patil and Kai 2010).

The details of function of many nuage proteins have been revealed by a series of recent studies. For example, Vasa interacts with UAP56 and likely functions for transport of piRNA precursors to the nuage (Zhang *et al.* 2012). The Tudor domain protein Krimp maintains Aub and Ago3 on the nuage for proper ping-pong amplification. Krimp interacts with Ago3, which does not require piRNA loading on Ago3 or Arginine methylation. However, the binding of Krimp to Aub requires arginine methylation of Aub. Krimp promotes arginine methylation on Ago3, and prevents the loading of antisense piRNA on it (Sato *et al.* 2015; Webster *et al.* 2015) (Figure 4). The nuage components Tej and Tap function synergistically for piRNA production, and are required for maintenance of all other piRNA pathway components at the nuage (Patil and Kai 2010; Patil *et al.* 2014). Another Tudor domain protein, Qin/Kumo, is also required for proper maintenance of Aub and Ago3 at nuage, and it prevents the loading of sense piRNAs to Piwi and Aub, thereby enforcing heterotypic Aub: Ago3 ping-pong (Zhang *et al.* 2011; Anand and Kai 2012; Wang *et al.* 2015). The function of piRNA pathway proteins explains, to some extent, the observed mutual dependence for their localization to nuage based on a genetic hierarchical relationship (Lim and Kai 2007; Anand and Kai 2012; Patil *et al.* 2014). For example, Vasa, which is genetically farther upstream, also becomes functionally upstream. Similarly, Krimp, Qin/Kumo, Tej, and SpnE support ping-pong, and, therefore, also support Aub and Ago3 on nuage. The piRNA biogenesis requires a multistep mechanism, and, as such, a number of proteins assist Aub and Ago3 for piRNA generation and piRNA-mediated degradation of transposon transcripts.

### piRNA-mediated transcriptional silencing of transposons

Transposon transcripts are post-transcriptionally silenced by piRNAs at the nuage, possibly at cytoplasmic processing bodies where piRNA pathway components are localized in later stages of oogenesis (Lim *et al.* 2009). In addition, piRNAs transcriptionally repress transposons, and piRNA loss leads to concomitant loss of repressive histone marks at transposons in the *Drosophila* female germline and somatic cells (Klenov *et al.* 2007, 2014; Sienski *et al.* 2012; Le Thomas *et al.* 2013; Rozhkov *et al.* 2013).

#### Piwi is a key mediator of transcriptional silencing:

The piRNA loading on Piwi is important for its entry in to the nucleus. Piwi-piRISC enters the nucleus to transcriptionally silence the transposons in both somatic and germ cells (Le Thomas *et al.* 2013; Rozhkov *et al.* 2013). Piwi loss does, indeed, cause an increase in RNA polymerase II occupancy at promoter regions of transposons, as well as reduction of H3K9me3 levels (Sienski *et al.* 2012; Czech *et al.* 2013; Huang *et al.* 2013; Le Thomas *et al.* 2013; Rozhkov *et al.* 2013; Klenov *et al.* 2014). Although the enrichment of Piwi at transposon loci has still not been established, predominant loading of antisense piRNAs to Piwi led to the speculation that Piwi-piRISC scans for nascent transposon transcripts to enforce transcriptional repression (Han *et al.* 2015; Homolka *et al.* 2015; Mohn *et al.* 2015; Sato *et al.* 2015; Senti *et al.* 2015; Wang *et al.* 2015; Webster *et al.* 2015). Notably, although Piwi is equipped with a slicer domain, this domain is dispensable for piRNA production and transposon silencing (Darricarrere *et al.* 2013).

#### Distinct components act together with Piwi:

Piwi-piRISC interacts with proteins to enforce transposon silencing. Recent work has identified several downstream components of Piwi-piRISC for transposon transcriptional silencing in both the germline and somatic cells. Many proteins have been shown to act downstream of Piwi-piRISC for H3K9me3 enrichment at transposon loci.

The double CHHC zinc finger protein, gametocyte-specific factor 1 (GTSF1), has been shown as a downstream Piwi-piRISC partner in both germline and somatic cells in the ovary (Dönertas *et al.* 2013; Ohtani *et al.* 2013). GTSF1 interacts with Piwi to establish H3K9me3 at the transposon loci for repression of transposons (Dönertas *et al.* 2013; Ohtani *et al.* 2013). The downstream nature of GTSF1 is suggested by unchanged piRNA levels upon GTSF1 loss, but transposon derepression profile and loss of H3K9me3 at transposon loci mimic what has been observed upon Piwi loss (Dönertas *et al.* 2013; Ohtani *et al.* 2013). However, the precise molecular mechanisms that explain how GTSF1 engages Piwi in TGS remain elusive. Another protein, Panoramix/Silencio (Panx), is vital for transposon repression through transcriptional silencing (TS) in germ cells (Sienski *et al.* 2015; Y. Yu *et al.* 2015). Panx interacts with Piwi and recruits the methyltransferase Egg to deposit H3K9me3 for heterochromatin formation at transposon loci. The loss of Panx leads to global transposon derepression without any reduction of piRNAs, suggesting its role as a downstream partner of Piwi-piRISC (Sienski *et al.* 2015; Y. Yu *et al.* 2015). In addition, Piwi-piRISC may silence transposons through the removal of H3K4me2 (Fadloun *et al.* 2013; Klenov *et al*. 2014). Depletion of the Lsd1 demethylase in *Drosophila* ovaries resulted in derepression of a subset of transposons, which is independent of Panx (Czech *et al.* 2013).

The function of Piwi-RISC for TGS of transposons is through not only histone modifications but also mediated by chromatin binding proteins. For example, Piwi loss leads to reduction of HP1a at few transposons in both germline and somatic cells in the ovary (Ohtani *et al.* 2013; Klenov *et al.* 2014). In germ cells, HP1a loss leads to derepression of transposons (Wang and Elgin 2011). However, in somatic cells, transposon derepression resulting from HP1a loss is not necessarily correlated with that caused by Piwi loss (Ohtani *et al.* 2013). This suggests that other chromatin regulators could repress some transposons independent of Piwi. Functions of these proteins also overlap, for example, HP1a likely enforces transposon silencing downstream of Piwi-piRISC by recruiting SetDB1 via interaction with Piwi-RISC tethered at the transposon loci (Brower-Toland *et al.* 2007; Sienski *et al.* 2015). Recently, histone H1 was also shown to be one of the downstream components of Piwi-piRISC for transposon repression in ovarian somatic cells, functioning in parallel with HP1a. However, unlike HP1a, H1 function is independent of H3K9Me3 marks (Iwasaki *et al.* 2016). Hence, it is possible that Piwi recruits different downstream factors, such as HP1a and H1, for more efficient transposon repression.

Current studies suggest that Piwi acts with different downstream factors to repress different sets of transposons. Although the underlying mechanism is not fully understood, the transposon location, type and evolutionary age are speculated to contribute to this. Indeed, the evolutionarily older transposons are shown to be enriched at pericentric regions and are targeted by fewer piRNAs (Kofler *et al.* 2012; Kelleher and Barbash 2013). It has been suggested that evolutionarily older transposons are more likely to be silenced transcriptionally, while the evolutionarily younger transposons depend more on the post-transcriptional silencing mechanism (Senti *et al.* 2015). In summary, the piRNA pathway incorporates a wide variety of partners to maintain piRNA clusters, transport, TGS and PTGS, for effective silencing of transposon globally and better sustainability of species in an evolutionary arms race with transposons.

## Mechanisms that Regulate Meiotic Cell Maturation

The maturation of both male and female gametes is a step-wise developmental process that requires the coordinated control of the cell cycle, cellular morphology, and cellular positioning. The coordination of these processes are absolutely essential, and require exquisite transcriptional, as well as translational, regulation of a diverse set of genes.

### Spermatocyte maturation

In the male germline, the transition from spermatogonia to spermatocytes is accompanied by a series of transcriptional, epigenetic, and morphological changes. After transit-amplification, germ cells undergo the last S phase followed by an extended G2 phase that initiates the spermatocyte stage. Spermatocytes grow 25 times in volume and turn on a robust transcription program to activate genes required for spermatocyte maturation, as well as genes needed for meiotic divisions and terminal differentiation (White-Cooper *et al.* 1998).

#### Transcriptional regulators:

Many genes required for meiotic divisions and terminal differentiation are under translational repression until a later time when their encoded proteins are required (Schafer *et al.* 1995). The G2/M transition in meiosis I requires *Cyclin B*, Boule (a RNA-binding protein) and Twine (Cdc25 homolog), all transcribed in spermatocytes (Alphey *et al.* 1992; Courtot *et al.* 1992; White-Cooper *et al.* 1998). Boule translocates from the nucleus to the cytoplasm to trigger the G2/M transition in meiosis I by allowing translation of Twine (Maines and Wasserman 1999). At this point in time, *Cyclin B* also escapes from translational repression and accumulates Cyc B protein in the cytoplasm of spermatocytes (White-Cooper *et al.* 1998). In both *boule* and *twine* mutant testes, spermatid differentiation occurs in a manner independent of meiotic cell cycle progression, suggesting that these two processes can be uncoupled (Alphey *et al.* 1992; Eberhart *et al.* 1996). However, the discovery of two classes of genes expressed in early spermatocytes reveals a high degree of coordination between meiotic divisions and spermatid differentiation (Lin *et al.* 1996). Mutations in any of these genes arrest meiosis and block spermatid differentiation, leading to testes filled with immature spermatocytes. These genes are named “meiotic arrest” genes, which are further classified into “*aly*-class” and “*can*-class” based on morphological differences of the chromosomal structure in the mutant spermatocytes (Lin *et al.* 1996; White-Cooper *et al.* 1998) and their distinct target genes (Lin *et al.* 1996; White-Cooper *et al.* 1998, 2000; Hiller *et al.* 2001, 2004; Ayyar *et al.* 2003; Jiang and White-Cooper 2003; Perezgasga *et al.* 2004; Beall *et al.* 2007; Jiang *et al.* 2007; Chen *et al.* 2011). For example, transcription of meiotic cell cycle genes, such as *Cyclin B*, *boule*, and *twine*, rely on *aly*-class, but not *can*-class, genes (White-Cooper *et al.* 1998). However, Boule protein accumulation requires the *can*-class genes (Chen *et al.* 2005). Since meiotic arrest genes regulate transcription or translation of meiotic cell cycle genes, the meiotic cell cycle cannot proceed until terminal differentiation genes are robustly transcribed (Lin *et al.* 1996; White-Cooper *et al.* 1998).

The six known *aly*-class genes are *always early* (*aly*), *cookie monster* (*comr*), *matotopetli* (*topi*), *tombola* (*tomb*), achintya/vismay (*achi*/*vis*), and *Caf1* (Beall *et al.* 2007). All of the *aly*-class genes, except *achi/vis*, are expressed exclusively in primary spermatocytes (Ayyar *et al.* 2003; Jiang and White-Cooper 2003; Wang and Mann 2003; Perezgasga *et al.* 2004; Jiang *et al.* 2007; White-Cooper 2009). Four *aly*-class proteins have putative DNA-binding domains, including Comr, which contains a winged helix; Topi, which contains multiple Zn-finger motifs; Tomb, which has a CXC domain; and Achi/Vis, products from a gene duplication, which have homeodomains. Thus, it is thought that these proteins regulate the transcription of target genes by directly binding to DNA sequences, even though their direct target genes have not been identified. Immunoaffinity purification studies have revealed that Aly and Tomb proteins are copurified with Mip40 (Myb interacting protein, 40 kDa) to form the testis meiotic arrest complex tMAC, which also contains Topi, Comr, and CAF1 (Beall *et al.* 2007). A second form of tMAC contains Aly, Comr, and Achi/Vis (Wang and Mann 2003). The tMAC resembles the MIP/dREAM complex in mammals and the SynMuv complexes in *C. elegans* (White-Cooper *et al.* 1998, 2000; Ayyar *et al.* 2003; Jiang and White-Cooper 2003; Perezgasga *et al.* 2004; Beall *et al.* 2007; Jiang *et al.* 2007). Studies using the DamID method profiled ∼300 direct target genes of Comr in testis, most of which have decreased expression in the *comr* mutant, suggesting that it functions mainly as a transcriptional activator (Laktionov *et al.* 2014). This is consistent with earlier results demonstrating that expression of Achi/Vis fused with a strong transactivation domain, VP16, rescued the *achi*/*vis* mutant phenotype, while the fusion of Achi/Vis with a repression domain, EnR, failed to rescue (Wang *et al.* 2008). Consistent with these findings, all tMAC subunits have been found to colocalize with euchromatin in primary spermatocytes (White-Cooper *et al.* 2000; Jiang and White-Cooper 2003; Wang and Mann 2003; Jiang *et al.* 2007).

The *can*-class genes encode testis-specific homologs of ubiquitously expressed subunits of the general transcription factor II D (TF_II_D). TF_II_D is one of the general transcription factors that constitute the RNA Pol II preinitiation complex composed of TATA-binding protein (TBP) and 13–14 TBP-associated factors (TAFs) (Tora 2002; Matangkasombut *et al.* 2004; Cler *et al.* 2009). TF_II_D coordinates the interaction between RNA Pol II and gene promoter regions. The characterized *can*-class genes include *cannonball* (*can*, *TAF5L*), *meiosis I arrest* (*mia*, *TAF6L*), *no hitter* (*nht*, *TAF4L*), *ryan express* (*rye*, *TAF12L*), and *spermatocyte arrest* (*sa*, *TAF8L*). Among the five TAF homologs, four, including Mia, Nht, Rye, and Sa, share similar structural domains called histone folding motifs for protein–protein interaction, while Can is a WD40-repeat-containing protein (Hiller *et al.* 2001). Indeed, Nht and Rye form a heterodimer *in vitro* (Hiller *et al.* 2004). These testis-specific TAFs (tTAFs) are thought to form a testis-specific complex required for transcriptional activation of the terminal differentiation genes (Hiller *et al.* 2001, 2004). Such predicted functions of tTAFs suggest that they localize at the euchromatin in spermatocyte nuclei. However, while a proportion of the total protein of each tTAF associates with chromosomes in spermatocytes, most tTAF protein is localized to a subcompartment within nucleolus (Chen *et al.* 2005; Metcalf and Wassarman 2007). Interestingly, Polycomb and other components of PRC1 are colocalized to the same nucleolar subcompartment with tTAFs in spermatocytes. Furthermore, localization of PRC1 components to the spermatocyte nucleolus is coincident with tTAF expression and dependent on wild-type tTAF function (Chen *et al.* 2005). These results suggest that tTAFs act as derepressors by sequestering PRC1 to the spermatocyte nucleolus to counteract PcG-induced repression. However, removing PcG activity is not sufficient to turn on terminal differentiation genes in the absence of tTAFs (Chen *et al.* 2011), suggesting that chromatin-associated tTAFs are required to activate terminal differentiation genes. Consistent with these observations, tTAFs were reported to turn on transcription of >1000 genes, many of which are required for spermatid differentiation (White-Cooper *et al.* 1998; Chen *et al.* 2011). Among the tTAF-dependent genes, three are shown to be direct target genes of tTAF by ChIP assay: *fuzzy onions* (*fzo*), which encodes a protein required for mitochondrial fusion in early spermatids (Hales and Fuller 1997); *mst87F*, which encodes a component of the sperm tail (Schafer *et al.* 1993) and *don juan* (*dj*), which encodes a sperm-specific DNA-binding protein that also localizes to mitochondria (Santel *et al.* 1998). ChIP analysis at the promoter regions of these three genes directly targeted by tTAF showed that levels of the repressive H3K27me3 mark and paused Pol II are high, while levels of the active H3K4me3 mark are low in *can* and *aly* mutant testes (Chen *et al.* 2011). These data suggest that tTAFs and tMAC might recruit TrxG, whose activities antagonize PcG, to methylate H3K4 at promoters of terminal differentiation genes and activate robust transcription (Chen *et al.* 2005).

Although the mode of interaction between tMAC components (*aly*-class) and tTAFs (*can*-class) is not fully understood, the transcription coactivator Mediator likely acts to coordinate tMAC and tTAFs. The tMAC recruits Mediator components to spermatocyte chromatin, and Mediator subsequently helps proper tTAF localization. Together, tMAC, tTAFs and Mediator coregulate a cohort of spermatid differentiation gene expression (Lu and Fuller 2015). It was also reported that the function of *aly* is required for the binding of TAF8L to target gene promoters. Aly is also required for the proper nucleolar localization of several tTAFs and Polycomb in spermatocytes, suggesting that tMAC acts upstream of tTAFs (Chen *et al.* 2011). This is consistent with assays using Northern blot, *in situ* hybridization, and microarray analysis (White-Cooper *et al.* 1998; Hiller *et al.* 2001; Chen *et al.* 2011). In addition, while Mip40 is coimmunoprecipitated with tMAC components, loss of *mip40* results in spermatocytes with condensed chromosomes, a phenotype similar to mutants of *can*-class genes (Beall *et al.* 2007), suggesting that Mip40 might mediate the interaction between tMAC and tTAFs. Both tMAC and tTAFs have their canonical counterparts that act generally in other tissues, as well as in spermatocytes, probably by regulating target genes distinct from the testis-specific forms. Similarly, the canonical chromatin remodeler NURF has a germline-specific function in regulating meiotic divisions and spermatocyte differentiation (Kwon *et al.* 2009), most likely through using an alternatively spliced isoform.

Five other meiotic arrest genes, which cannot be classified as either *aly*-class or *can*-class, were identified and characterized. Wake-up-call (Wuc) was identified by its physical interaction with Aly in a yeast-two-hybrid screen (Jiang *et al.* 2007). In spermatocytes, the Wuc protein is highly expressed and associated with chromatin, similar to other tMAC components. However, unlike tMAC or tTAF mutants, loss of *wuc* does not abolish expression of either meiotic cell cycle genes or spermatid differentiation genes (Doggett *et al.* 2011). Another study showed that disruption of a component of the THO complex, THOC5, led to the meiotic arrest phenotype. The THO complex is known to export mRNAs from nucleus to cytoplasm. However, no mRNA export defects were detectable in the *thoc5* mutant. Moreover, neither meiotic cell cycle genes nor spermatid differentiation genes have decreased transcription in the *thoc5* mutant, even though a more comprehensive study is needed. THOC5 is localized to a perinucleolar region, and loss of *thoc5* function leads to disrupted nucleolar structure and the localization of tTAFs, which could contribute to its mutant phenotype (Moon *et al.* 2011). A more recent study identified Ntx1, another mRNA export machinery component, as required for accumulation of many spermatogenesis-specific mRNAs. However, the dependence of these transcripts on Ntx1 has a distinct mode compared to tMAC- or tTAF-dependent genes, which is regulated by the primary transcript structure (Caporilli *et al.* 2013). Moreover, through characterization of a meiotic arrest mutant *magellan* (*magn*), the *Ubi-p63E* gene encoding polyubiquitin has been shown to regulate proper spermatocyte chromatin structure, meiotic cell cycle progression, and spermiogenesis. However, the different phenotypes caused by loss-of-function of proteasome subunits suggest that Ubi-p63E acts in a protein degradation-independent manner in spermatocytes (Lu *et al.* 2013). Finally, a very recent study identified a novel meiotic arrest gene *kumgang* (*kmg*), which encodes a zinc finger-containing protein. The *kmg* gene is specifically turned on in early spermatocytes, independent of either tMAC or tTAF. Interestingly, Kmg is required to maintain germline identity by suppressing the expression of hundreds of somatic genes. Genetic, genomic, and biochemical analyses reveal that Kmg acts with the chromatin remodeler dMi-2 to restrict the tMAC component Aly from helping to fire transcription from cryptic promoters of a cohort of somatic genes, which are normally turned on in somatic tissues, such as gut and brain (Kim *et al.* 2017). Both identification of *wuc*, *thoc5*, *Ntx1*, *magn*, and *kmg* mutants, and detailed characterization of their phenotypes and mechanisms demonstrate the existence of meiotic arrest genes other than *aly*- and *can*-class. Further understanding of their molecular and cellular mechanisms will lead to new information about spermatocyte maturation.

#### MicroRNAs:

In males, *bam* mRNA is detectable, but Bam protein is undetectable in the meiotic spermatocytes. It has been shown that a specific miRNA, *miR-275*, represses Bam protein accumulation through *bam* 3′UTR in spermatocytes. If this repression of Bam protein accumulation in spermatocytes is misregulated, spermiogenesis cannot proceed properly, and this will lead to decreased male fertility (Eun *et al.* 2013). Therefore, although Bam is an important differentiation factor to initiate GSC differentiation, its downregulation is also critical for proper spermatid terminal differentiation. Furthermore, this post-transcriptional regulation of Bam protein accumulation does not occur in the female germline, again suggesting sex-specific modes in the regulation of meiotic germ cell maturation.

### Oocyte specification and maturation

Oocyte development begins with oocyte specification in the germarium; once specified, the oocyte migrates to the posterior region of the cyst, which will be enclosed by follicle cells and buds off as an egg chamber. While the egg chamber is growing, the polarity of the oocyte is established. These processes involve both signal transduction pathways, as well as the cytoskeletal machinery. Furthermore, the piRNA pathway has also been reported to have a significant role in establishing oocyte polarity and proper oocyte development.

#### Oocyte specification and polarity establishment in the germarium:

As an extremely specialized cell type, oocyte formation requires a series of developmentally regulated processes to break the symmetry and give rise to this highly polarized, gigantic cell (Roth and Lynch 2009). Polarity formation begins in the germarium with the specification of oocytes, and this polarity is already established as early as the first division of the CB (de Cuevas and Spradling 1998). In regions 2a and 2b (Figure 1A), mRNA transport allows accumulation of specific markers in the developing oocyte (Suter and Steward 1991; Lantz *et al.* 1994; Mach and Lehmann 1997). After completing the transit-amplification stage, the microtubule organizing center (MTOC) appears in one or two cells inside the 16-cell cyst, which has four ring canals connected to other cells in the cyst. These two cells, called pro-oocytes, start meiosis, which is characterized by the appearance of double-strand breaks (DBSs) and the formation of synaptonemal complex (SC). Later, one of them will be committed to an oocyte. Upon the specification of oocyte, DBSs, which have been seen in both pro-oocytes, become restricted to one oocyte in region 2a/2b, and are repaired thereafter (Jang *et al.* 2003). In piRNA pathway mutants, such as *armitage* (*armi*) and *aub*, DSBs persist longer and are accumulated in the oocyte during later stages of development (Klattenhoff *et al.* 2007). The upregulation of transposons, and their increased transpositions in the oocyte, are believed to cause accumulation of DSBs in the oocyte, although no study has explicitly proven this. At region 2b/3 (Figure 1A), oocyte determination is evident in that the SC remains in only one germ cell determined to be an oocyte (Huynh and St Johnston 2000; Page and Hawley 2001). In addition, MTOC appearance is more pronounced, and *gurken* (*grk*) mRNA is localized posteriorly (Neuman-Silberberg and Schupbach 1993, 1996). Egg chambers (Stage 1–14) are assembled posterior to the germarium, which contains the nurturing nurse cells and the developing oocyte ensheathed by follicle cells derived from FSCs.

#### Oocyte determination and polarity formation during oocyte maturation:

As oogenesis proceeds toward region 3, the oocyte meiotic chromosomes form a compact spherical structure called the karyosome, which is mainly transcriptionally silent (Parfenov *et al.* 1989; Bastock and St Johnston 2008; Lancaster *et al.* 2010). At this stage, the oocyte development is also determined by the coordinated activity of both cell cycle genes and polarity genes (Lilly and Spradling 1996; Mach and Lehmann 1997; Mata *et al.* 2000; Huynh *et al.* 2001a; Hong *et al.* 2003).

Microtubule networks, as organized by spectrosome/fusome structure (Grieder *et al.* 2000), play important roles in the formation and maintenance of oocyte polarity. The MTOCs shift from the anterior to the posterior side of the oocyte (Theurkauf *et al.* 1992, 1993; Huynh *et al.* 2001b; Vaccari and Ephrussi 2002). The reorganized microtubule network is important for proper localization of maternal components, such as *bicoid* and *oskar* (*osk*), as polarity determinants, and define the anterior and posterior sides of the developing oocyte, respectively (Ephrussi *et al.* 1991; Kim-Ha *et al.* 1991; Brendza *et al.* 2000; Januschke *et al.* 2002). This polarity within the oocyte also defines the embryonic anterior−posterior axis. Live imaging of Osk particle movement during oogenesis showed that the mRNA is actively transported along microtubules in all directions, with a slight bias toward the posterior (Zimyanin *et al.* 2008). Meanwhile, *grk* mRNA is found at the posterior of oocyte in the germarium, while in the later stages, *grk* mRNA is repositioned at the dorsal anterior corner of the oocyte (Neuman-Silberberg and Schupbach 1996; Van Buskirk and Schupbach 1999; St Johnston 2005). Grk accumulation defines the dorsal−ventral axis of the oocyte, as well as the embryos (Schupbach 1987; Neuman-Silberberg and Schupbach 1993; Nilson and Schupbach 1999; Moussian and Roth 2005). Grk localization and oocyte nucleus migration are mediated by microtubules (reviewed by Roth and Lynch 2009). Although the exact mechanism for *grk* mRNA localization is not known, microtubules from MTOC and dynein are important for Grk localization in the oocyte and mediate communication with follicle cells (Brendza *et al.* 2000, 2002; Duncan and Warrior 2002; Januschke *et al.* 2002). Together, polarized localization of *bicoid*, *osk*, and *grk* mRNAs defines both the AP and DV axes of the oocyte and embryo. However, imaging data revealed that the overall microtubule network is actually much less polarized than previously expected (MacDougall *et al.* 2003; Zimyanin *et al.* 2008), provoking more studies using new techniques such as live cell imaging.

By midoogenesis, a specialized cytoplasm, termed as pole plasm, assembles at the posterior end of oocyte (Hay *et al.* 1988; Lasko and Ashburner 1988; Ephrussi *et al.* 1991; Golumbeski *et al.* 1991; Ephrussi and Lehmann 1992; Harris and Macdonald 2001;). The pole plasm contains granules of ribonucleoproteins (RNPs) enriched with RNAs and proteins, which are required for formation of primordial germ cells (PGCs; Santos and Lehmann 2004). The pole plasm is maternally transmitted to the embryo and retained at the posterior side, where the nuclei are first cellularized to form the primordial germline cells (Starz-Gaiano and Lehmann 2001; Santos and Lehmann 2004; Laver *et al.* 2015). As previously noted, piRNAs are also deposited maternally to the embryos, conferring defense against transposons in the next generation (Brennecke *et al.* 2008). In addition to piRNAs, several piRNA pathway components, such as Piwi, Vasa, Tud, and Aub, are found in the pole plasm and required for pole plasm formation (Hay *et al.* 1988; Lasko and Ashburner 1988; Ephrussi *et al.* 1991; Golumbeski *et al.* 1991; Ephrussi and Lehmann 1992; Harris and Macdonald 2001; Megosh *et al.* 2006).

#### Function of PcG in oocyte specification:

While the determined oocyte will initiate extraordinary cell growth and meiotic cell cycle, the 15 nurse cells in the *Drosophila* ovary will enter the endocycle and become polyploid cells to provide RNAs and proteins to the developing oocyte. Transdetermination from oocyte to nurse-like cells was observed when PRC2 components *E*(*z*) and *Su*(*z*)*12* were knocked down in the *Drosophila* female germline. This cell fate change results from derepression of Cyclin E and cyclin-dependent kinase inhibitor Dacapo upon loss of the repressive H3K27me3 mark (Iovino *et al.* 2013). However, such cell fate switch does not occur in the male germline. In males, all 16 spermatogonial cells enter meiosis simultaneously after mitosis and differentiate into mature sperm synchronously. This phenomenon suggests that differences between the female and male germline differentiation pathways require distinct epigenetic regulators.

#### piRNA pathway components for polarity formation:

In addition to cytoskeletal machinery and RNA-binding proteins, loss of many piRNA pathway proteins results in discernible defects in polarity formation. For example, in *armi*, *spnE*, *zuc*, *mael*, and *krimp* mutants, Grk and Osk proteins fail to localize to the dorsal–anterior region and to the posterior region, respectively (Findley *et al.* 2003; Cook *et al.* 2004; Chen *et al.* 2007; Klattenhoff *et al.* 2007; Lim and Kai 2007; Pane *et al.* 2007). Failure of microtubule network polarization in piRNA pathway mutants is believed to cause mislocalization of these components. In addition, Osk is precociously translated in some of the piRNA pathway mutants (Cook *et al.* 2004; Lim and Kai 2007; Pane *et al.* 2007). The piRNA pathway component Mael interacts with MTOC components, including centrosomin, mini spindles, and γ−tubulin (Sato *et al.* 2011). The interaction of Mael with cytoskeletal structure further strengthens the role of piRNA pathway components in oocyte polarity formation. Interestingly, upregulation of *I-element* transposon is known to perturb the localization of *grk* and *bcd* mRNAs (Van De Bor *et al.* 2005). However, some piRNA pathway mutants, such as *tej*, and *qin/kumo*, do not show defects in the localization of *grk* or polarity formation of oocyte, despite the severe depression of transposons including *I-element* (Patil and Kai 2010; Anand and Kai 2012; Patil *et al.* 2014), suggesting that derepression of transposons alone is not sufficient to cause polarity defects in oocytes.

Consensus has still not formed around the role of piRNA pathway proteins in DNA damage response or polarity determination. In some piRNA pathway mutants, ablation of DNA damage checkpoint components, such as *mei41* and *chk2*, could suppress oocyte polarity defects (Klattenhoff *et al.* 2007), suggesting crosstalk between some microtubule polarization components and the DNA damage response pathway. In contrast, Grk mislocalization in *mael*, *squ*, and *zuc* mutants could not be restored by ablating *mei41* function (Pane *et al.* 2007; Sato *et al.* 2011). Therefore, either DNA damage response in those mutants is initiated by a different downstream component or a different DNA damage-sensing pathway is involved. Except for Mael, no other piRNA pathway protein is known to directly interact with cytoskeleton components (Sato *et al.* 2011). Under these circumstances, the effect on polarity formation is a direct or indirect effect of loss of these proteins.

## Mechanisms in Regulating Intergenerational and Transgenerational Epigenetic Inheritance

Traditionally, heritability is a characteristic feature of the genetic material of an organism, notably its DNA. Nonetheless, many phenomena and mechanisms of non-DNA sequence-based inheritance of vastly different phenotypes have been described from one generation to the next in multiple organisms ranging from plants to vertebrates (Youngson and Whitelaw 2008; Heard and Martienssen 2014). This inheritance of information beyond the primary DNA sequence is known as epigenetic. Direct epigenetic inheritance from parent to offspring is termed intergenerational epigenetic inheritance (IEI), and it is distinguished from transgenerational epigenetic inheritance (TEI), which is observed in generations that were not exposed to the initial signal or environment that triggered the acquired change.

One of the earliest reports of TEI in *Drosophila* was uncovered studying the Fab-7 chromosomal boundary element (Cavalli and Paro 1998). The Fab-7 boundary element, also a member of Polycomb Response Elements, is derived from the bithorax complex (BX-C) and is required to prevent crosstalk between adjacent regulatory regions, *iab-6* and *iab-7*, which control the spatial expression of the *Abd-B* gene (Hagstrom *et al.* 1996). To determine the function of the defined Fab-7 element, transgenic reporter strains were engineered to carry the Fab-7 element upstream of a GAL4 UAS-inducible *lacZ* reporter and a *mini-white* gene. The Fab-7 element was found to act as a strong silencer, repressing expression of both *lacZ* and the distantly located *mini-white* gene (Zink and Paro 1995). Increased GAL4 expression could stably activate both *lacZ* reporter and *mini-white* gene. Furthermore, a short, single pulse of GAL4 expression, regulated by a heat-shock promoter (hs-GAL4), during embryogenesis was sufficient to induce activation of the *mini-white* gene throughout development, resulting in adult flies with red eyes (Cavalli and Paro 1998). This continued expression of the reporter suggests a loss of silencing that is mitotically inheritable over many cell divisions and not dependent on the duration of the GAL4 protein. Surprisingly, GAL4-independent transmission of the active *mini-white* gene could be propagated through the female gametes for four generations. Inheritance of the expression pattern was not observed in the male germline. This was tested under conditions where offspring did not inherit the *hs-GAL4*, demonstrating that a short pulse of GAL4 induced during early embryogenesis alters the epigenetic landscape of a gene in a way that is stably inherited during both mitosis and meiosis. The molecular carrier for the maintenance of these patterns of expression through meiosis remains to be determined. Despite the fact that the mechanistic basis underlying both IEI and TEI is largely unknown and under intense investigation, three epigenetic information carriers have, in fact, been identified, including DNA methylation, chromatin structure, and RNAs.

### DNA methylation in intergenerational and transgenerational epigenetic inheritance

DNA methylation may function as a molecular carrier during IEI in *Drosophila*. Inheritance of 5mC DNA methylation has been well documented in both mammalian and plant models of epigenetic inheritance (Heard and Martienssen 2014). Although the full extent to which DNA methylation participates in IEI in *Drosophila* remains elusive, investigations of sister chromatid inheritance, as well as tumor susceptibility, have highlighted two separate cases of intergenerational epigenetic effects in genetically compromised backgrounds.

During ACD of male GSCs, sister chromatids of the X and Y chromosome are distinguished and segregated in a nonrandom manner (Yadlapalli and Yamashita 2013). Loss of *DNMT2* leads to randomized sister chromatid segregation of both X and Y chromosomes, suggesting that *DNMT2* confers epigenetic information to the X and Y chromosomes that leads to distinct sister chromatid segregation. Furthermore, systematic crosses between homozygotic and heterozygotic *DNMT2* parents revealed that parental DNMT2 function is necessary for proper segregation of X and Y sister chromatids in the next generation in a parent-dependent manner, very much like the imprinting phenomenon. These data suggest that parental DNMT2 functions during gametogenesis in both males and females to transmit heritable information on the X and Y chromosomes, and such information is maintained during early embryogenesis (Yadlapalli and Yamashita 2013).

Little is known about epigenetic reprogramming of DNA methylation during gametogenesis and early embryogenesis in *Drosophila*. Studies of an oncogenic JAK kinase encoded by *hopscotch^tum-1^* (*hop^tum-1^*) demonstrated that this temperature-sensitive hypermorphic allele is able to antagonize a cellular program that erases DNA methylation of parental origin, allowing the epigenetic alterations to be maintained in the absence of the original mutation (Xing *et al.* 2007). Tumorous-lethal (Tum-1) is a dominant temperature-sensitive mutation in the *hop* locus that leads to overproliferation of hemocytes and formation of melonotic tumors, which are black masses of hemocytes correlated with lethality. In a genetic approach to identify genes important for *hop^tum-1^*-induced tumorigenesis, 37 modifiers that either enhanced or suppressed *hop^tum-1^* tumorigenesis were identified (Shi *et al.* 2006). Interestingly, many of the identified mutations exhibited paternal-effect modification of *hop^tum-1^* tumor susceptibility. For example, one of the modifiers, *Kruppel* (*Kr*), enhances *hop^tum-1^* tumorigenicity. When *hop^tum-1^* heterozygotic females were mated with male heterozygotes for *Kr*, tumorigenesis associated with *hop^tum-1^* was enhanced in the F1 generation, irrespective of the inheritance of the modifier mutation itself. This enhancement persisted into the F2 generation, but diminished by the F3 generation. Further studies indicate that the *Kr* mutation establishes DNA methylation at promoters during early embryogenesis. Furthermore, the epigenetic alterations induced by *Kr* are normally erased in the next generation. However, in the presence of the *hop^tum-1^* allele, the increased DNA methylation induced by *Kr* was transmitted to the next generation.

### Chromatin structure in intergenerational and transgenerational epigenetic inheritance

Histones and histone variants have become primary candidates for mediating germline epigenetic inheritance. Histone modifications and variants are capable of transmitting epigenetic information through mitosis and meiosis to the next generation (Gaydos *et al.* 2014). A major barrier to IEI or TEI is the epigenetic reprogramming during gametogenesis and early embryo development, during which global changes in histone modifications and variants occur (Harrison and Eisen 2015). An extreme example of histone replacement is the transition from nucleosome-based to protamine-based chromatin structure during *Drosophila* spermatogenesis (Rathke *et al.* 2007). Upon fertilization, chromatin undergoes dramatic remodeling again when the paternal genome is remodeled, replacing protamines with the histone variant H3.3 (Loppin *et al.* 2005). The *Drosophila sesame* mutant exhibits lesions at the *HIRA* gene encoding H3.3/H4 replication-independent nucleosome assembly chaperone. Characterization of *sesame* revealed that protamines are replaced by maternal H3.3 prior to the first S phase during embryogenesis. Upon fertilization, H3.3 is used to remodel paternal chromatin. Despite these sweeping changes, recent proteomic analysis of whole sperm mass spectrometry has revealed that all four canonical histones, as well as histone variants, are retained in mature sperm (Dorus *et al.* 2006). This retention in mature sperm raises the possibility that they retain epigenetic information for transmission across generations.

The centromere-specific histone variant Centromere identifier (Cid) is also present in mature sperm (Raychaudhuri *et al.* 2012). In *Drosophila*, nucleosomes with Cid, instead of other histone H3 variants, are stably incorporated exclusively at the centromeric region. Analysis of centromere identity has indicated that the centromere is specified epigenetically (Black and Cleveland 2011). Cid is retained in mature sperm and during the protamine-to-histone transition after fertilization. This paternally inherited Cid is required for the maintenance of paternal chromosomes in the next generation. In the absence of paternally inherited Cid, paternal chromosomes fail to recruit the maternally provided Cid and cannot generate functional kinetochores during the first mitosis.

Paternal genome stability in the embryo also relies on a heterochromatin-associated protein 1 (HP1) paralog, HP1E. The *Drosophila* genome encodes five HP1 paralogs, HP1A–E. The genes that encode HP1A, HP1B, and HP1C are expressed in all tissues and localize primarily to heterochromatin, with the exception of HP1C, which localizes exclusively to euchromatin (Smothers and Henikoff 2001). HP1D and HP1E have special roles in the female and male germline, respectively (Volpe *et al.* 2001; Vermaak and Malik 2009). HP1D is required for transposon silencing in the female germline, and loss of HP1D results in female sterility (Volpe *et al.* 2001; Klattenhoff *et al.* 2009). Expression of HP1E is developmentally restricted within the male germline where it localizes to the developing spermatids and functions in heterochromatin integrity. Specifically, HP1E localizes to developing spermatids subsequent to the completion of Meiosis II, but it is not detectable in mature sperm. In the absence of HP1E in males, embryos have defects in paternal chromatin condensation and fail to separate chromosomes during mitosis, resulting in “chromatin bridges” and lethality (Levine *et al.* 2015). Unlike Cid, which is inherited from the previous generation, HP1E is not inherited from sperm; instead, HP1E primes paternal chromosomes during spermatogenesis to ensure proper segregation in the next generation.

### Role of piRNA pathway in maternal deposition of transcripts and their clearance

The oocyte provides transcripts and proteins to embryos for their early development. Transcripts of many genes, including those required for early development, are maternally deposited through the ring canals from nurse cells to the oocyte during oogenesis (reviewed by Laver *et al.* 2015). Transcription from zygotic genome starts at 2 hr postfertilization, and concurrently, a subset of maternally deposited materials is eliminated. This process is referred to as Maternal to Zygotic Transition (MZT; reviewed by Laver *et al.* 2015). During MZT, the transition of gene expression is tightly regulated in several different ways, including clearance of the maternally deposited transcripts. Recent high-throughput analyses identified that a significant number of transcripts, from 7000 to 10,000, are maternally transmitted to embryos (Lecuyer *et al.* 2007; Thomsen *et al.* 2010). Approximately two-thirds of them are either degraded, or significantly reduced, within 3 hr postfertilization (Thomsen *et al.* 2010; Laver *et al.* 2015). An RNA-binding protein, Smaug, triggers degradation of those RNAs by deadenylation through the CCR4/POP2/NOT4 deadenylase complex. Several studies revealed that piRNA pathway proteins and piRNAs promote the decay of a subset of posteriorly localizing maternally deposited RNAs in the bulk of embryo, possibly via the deadenylation complex, leading to the enrichment of germline determinants at pole plasm (Rouget *et al.* 2010; Barckmann *et al.* 2015; Vourekas *et al.* 2016).

#### Enrichment of *nos* transcript at pole plasm:

Simonelig and colleagues first reported that piRNA pathway proteins and piRNAs promote the deadenylation and decay of maternally deposited *nanos* (*nos*) transcripts (Rouget *et al.* 2010). Maternally deposited *nos* mRNA is present throughout embryos at very early stages, but it is translationally repressed in the somatic part and degraded in a deadenylation-dependent manner involving Smaug (Dahanukar and Wharton 1996). Osk, the key component of pole plasm formation, prevents deadenylation of *nos* transcript, and promotes its translation at the posterior pole, which helps to form Nos gradient at the posterior region (Santos and Lehmann 2004; Zaessinger *et al.* 2006). In addition to Osk, the piRNA pathway components *aub*, *ago3*, *spnE*, and *piwi* were shown to be required for deadenylation and decay of the maternal *nos* transcript (Rouget *et al.* 2010). Aub and Ago3 are present throughout embryos and likely trigger deadenylation of *nos* mRNA by recruiting the deadenylation complex. The piRNAs arising from *412* and *roo* transposons target *nos* 3′UTR for *nos* mRNA decay. Those piRNAs bound to Aub and Ago3 likely recruit Smaug and the CCR4-NOT adenylation complex to *nos* transcript and degrade it in the bulk embryo, but not at the pole, forming the Nos gradient (Rouget *et al.* 2010).

#### Decay and anchoring of maternal transcripts *en masse*:

Recent genome-wide analyses of Aub-bound RNAs using CLIP (crosslinking and immunoprecipitation) experiments by two groups further revealed the functions of piRNAs in anchoring and enriching maternally deposited transcripts at the posterior pole (Konig *et al.* 2010; Barckmann *et al.* 2015; Vourekas *et al.* 2016). While both small RNAs and long RNAs are found in the Aub-CLIP libraries, the small RNAs, mostly comprised of piRNAs, are more abundant than long ones. Almost all Aub-bound long RNAs bind to Aub in a piRNA-dependent manner, and do not contain transposon sequences, suggesting that RNAs bound to Aub are unrelated to piRNA biogenesis (Barckmann *et al.* 2015).

Both studies also reported that Aub-bound transcripts are derived from genes involved in diverse functions (Barckmann *et al.* 2015; Vourekas *et al.* 2016). Posterior localization of Aub does not seem to be necessary for binding with these transcripts, except for some localized at the posterior pole (Barckmann *et al.* 2015; Vourekas *et al.* 2016). A large number of posteriorly localizing transcripts, including *osk*, *germ cell-less* (*gcl*), *polar granule component* (*pgc*), *hsp83*, and *nos*, depend on Aub for their degradation in the bulk of embryos (Barckmann *et al.* 2015; Vourekas *et al.* 2016). Many of them are degraded by the deadenylation-dependent pathway because they are stabilized in the embryos with mutations of deadenylation complex components (Rouget *et al.* 2010; Barckmann *et al.* 2015). In addition, Simonelig and colleagues found very few secondary piRNAs pairing with Aub-bound transcripts, suggesting that the ping-pong mechanism contribute to the clearance of fewer maternal transcripts (Barckmann *et al.* 2015). Hence, Aub is likely acting for MZT by its endonucleolytic activity and through the deadenylation-dependent pathway. It is possible that Smaug and the downstream components are involved in degradation of these transcripts, possibly triggered by piRNAs.

Extensive computational analysis by Zissimos and colleagues further elucidated the importance of piRNAs in anchoring transcripts to Aub in embryos. Aub-bound piRNAs exhibit rather weak complementarity to mRNA in a manner reminiscent of miRNA–mRNA interaction (Vourekas *et al.* 2016). The transcripts localized at the posterior pole are enriched with such piRNA binding sites, suggesting that the Aub-piRNA complex in the pole granules may serve as nucleation sites for the proper localization of these transcripts. It is speculated that the same Aub-piRNA complex could also scan the transposon transcripts to maintain the fitness of species in the next generation.

Overall, piRNAs are important for maintaining mRNAs in Aub-RNA complexes. Aub functions to degrade transcripts of germline determinants in the bulk of cytoplasm, possibly by either endonucleolytic activity of Aub itself or triggering deadenylation, in turn leading to their enrichment in the pole plasm. In addition, Aub-bound piRNAs play an important role in anchoring the transcripts involved in posterior localization and germline development. Further studies are needed to elucidate the molecular mechanism that underlies the spatiotemporal regulation of Aub-piRNA complex/mRNAs interaction.

## Perspectives

**Figure 5:**
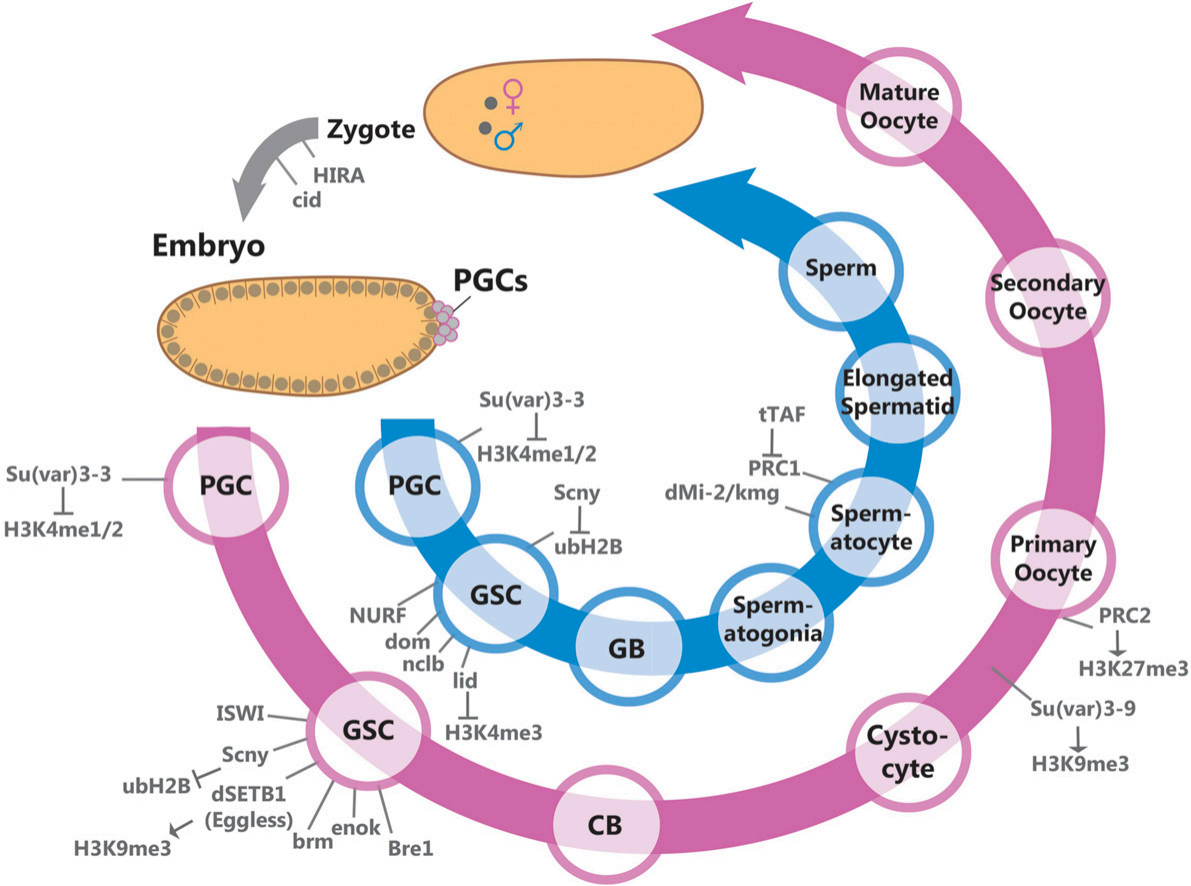
Epigenetic regulation of germ cell development in *Drosophila*. Male spermatogenesis (Blue arrow) and female oogenesis (Pink arrow) are highlighted at distinct differentiation stages (circles). Chromatin regulators, histone modifications, histone variants, and histone modifying enzymes are placed along the developmental timeline at specific stages in which previous studies have uncovered a critical developmental role. Schematic drawings of the zygote and early embryo include female (Venus symbol) and male (Mars symbol) pronuclei (gray) marked in the zygote, as well as PGCs, marked in the early embryo. Detailed information of these developmental mechanisms are discussed throughout the text.

In summary, to understand both oogenesis and spermatogenesis, developmental genetics and cell biology approaches can take advantage of the reliable developmental processes and distinct morphologies for each stage of germ cell differentiation. Formation of both male and female gametes requires the interaction between the germline and the somatic gonadal cells. During this process, the germline identity is protected, while GSC self-renewal, differentiation, and meiotic cell cycle genes are tightly regulated by the sequential changes of the chromatin structure in germ cells (Figure 5). On the other hand, mature gametes carry both genetic and epigenetic information from one generation to the next.

As described in this review, the germline genome must be protected against transposable elements (TE), or transposons, which are DNA sequences that can alter the genetic identity of a cell by changing their position in the genome. Charged with this task is the piRNA pathway, which is directly involved with the silencing of transposons. Both the steady state of TE repression, and the dynamics of the piRNA pathway during germline development are better understood today. Newly introduced transposons initially escape from repression via the piRNA pathway, but germ cells quickly acquire adaptation to new invasion of transposons by producing piRNAs in a single generation (Khurana *et al.* 2011). Repression of evolutionarily older transposons needs fewer piRNAs, while recent transposon insertions attract a higher number of piRNAs (Kelleher and Barbash 2013). Certainly, the study of piRNA pathway function from a population-wide perspective might shed light on the evolutionary nature and adaptive events in the piRNA population over generations. While studies have provided a glimpse of piRNA precursor transcription and processing, a proper understanding of events prior to piRNA processing remain enigmatic.

In the future, we can expect a better understanding of the unique features of germ cells, which will greatly facilitate applying them for the treatment of diseases and regenerative medicine. For example, piRNA pathway proteins are reported for transposon repression of embryonic stem cells in mammals (Darricarrere *et al.* 2013; Marchetto *et al.* 2013; Peng *et al.* 2016). Recently, the piRNA pathway proteins have also been shown to support survival and proliferation of cancer cells from flies to human (Janic *et al.* 2010; Fagegaltier *et al.* 2016; Ng *et al.* 2016; Sumiyoshi *et al.* 2016). In higher vertebrates, PIWIs are implicated in somatic stem cell functions and/or regeneration of the tissues (Rizzo *et al.* 2014). Based on the conserved nature of the piRNA pathway across species and during development and disease, understanding the relationship between piRNAs and transposons during early development may provide insight into the development of tumors, highlighting the importance of studying noncoding RNA regulation, and in turn, leading to the identification of new therapeutic targets.

However, it remains challenging to study epigenetic regulation at the individual gene in germ cells at particular stages, such as PGCs in embryos and GSCs in adults. A major technical hurdle involves obtaining a sufficient number of homogeneous cells to investigate their chromatin structure. However, technological advances have significantly reduced the required cell number for such studies, thus providing unprecedented opportunities to understand germ cell identity and activity. This step will be invaluable for treating diseases associated with defects in germ cell differentiation, such as infertility and germ cell tumors, as well as applying germ cells in regenerative medicine. Also, new imaging techniques, such as live cell imaging and superresolution imaging, in combination with genomic engineering, will allow us to trace distinct molecules, such as mRNAs and proteins, as well as organelles and subcellular structures in order to gain new insights into germ cell differentiation at individual developmental stages (Cheng *et al.* 2008, 2011; Sheng *et al.* 2009; Morris and Spradling 2011; Spradling 2011; Lenhart and DiNardo 2015; Shalaby and Buszczak 2017). Furthermore, new advances in genomic analyses, including RNA-seq, ChIP-seq, and Hi-C, are beginning to reveal chromatin structure during germline development in a sequence-specific manner. We anticipate rapid progress in the near future to resolve dynamic epigenetic regulation of germ cell differentiation at single-cell resolution, in real-time, and at both genomic and specific gene loci in *Drosophila*.
